# The epigenetics effects of transposable elements are genomic context dependent and not restricted to gene silencing in *Drosophila*

**DOI:** 10.1186/s13059-025-03705-4

**Published:** 2025-08-18

**Authors:** Marta Coronado-Zamora, Josefa González

**Affiliations:** Institut Botànic de Barcelona, CSIC, CMCNB, Barcelona, Spain

**Keywords:** H3K9me3, H3K27ac, Histone marks, *Drosophila*, Gene expression, Tissue specificity

## Abstract

**Background:**

Transposable elements (TEs) represent a threat to genome integrity due to their proliferative capacity. Eukaryotic cells silence TEs through different molecular mechanisms, including the deposition of repressive histone marks. Previous studies have shown that TE repressive marks can spread to neighboring sequences. However, evidence for this spreading leading to nearby gene silencing remains limited. Similarly, whether TEs induce changes in the enrichment of active histone marks genome-wide, and the potential impact on gene expression have not been widely studied.

**Results:**

In this work, we perform a comprehensive study of the epigenetic effects of 2235 TEs and their potential effects on nearby gene expression on *Drosophila melanogaster* head, gut, and ovary. While most TEs (816) induce the enrichment of the H3K9me3 repressive mark, with stronger epigenetic effects in the ovary, a substantial number (345 TEs) induce the enrichment of the H3K27ac active mark, particularly in the gut. We find that 70% of the H3K9me3 enriched TEs associated with expression changes downregulate the nearby gene, and 50% of the H3K27ac enriched TEs associated with expression changes lead to gene upregulation. These changes in expression affect specific regulatory networks only in the head. Furthermore, TE epigenetic effects on gene expression are genomic context dependent. Finally, we find that 221 TEs also affect gene expression by disrupting regions enriched for histone marks.

**Conclusions:**

Overall, our results show that TEs contribute to the generation of regulatory novelty through epigenetic changes, with these epigenetic effects not restricted to gene silencing and being genomic context dependent.

**Supplementary Information:**

The online version contains supplementary material available at 10.1186/s13059-025-03705-4.

## Background

The capacity of transposable elements (TEs) to move and expand within the genome is a threat to genome integrity [1, 2]. As a result, cells have evolved a range of molecular strategies, including epigenetic mechanisms, to repress and control TE activity [3, 4]. One epigenetic mechanism widespread among eukaryotes is the methylation of H3K9 (H3K9me3), a repressive mark associated with the formation of heterochromatin, that targets TEs to suppress their activity [5–8]. While the enrichment of repressive epigenetic marks at TEs would limit the expression of TEs, which should be beneficial for the cell, several studies have shown that TE silencing could also influence nearby gene sequences across species, including plants [9–11] and animals [6, 12, 13]. A potential functional consequence of the spread of repressive marks is the downregulation of the expression of nearby genes. However, the evidence for this effect genome-wide is limited across species [1, 14]. Several works have used *Drosophila* species to investigate the potential effect of TE repressive marks on nearby gene expression. In *D. albomicans*, TEs located nearby lowly expressed genes showed a higher H3K9me3 enrichment and spreading than those near highly expressed genes [15]. However, the lack of systematic downregulation between genes with insertions and their orthologs, lacking the insertions, suggests that TEs preferentially accumulate near lowly expressed genes rather than affecting their expression. The few studies comparing the expression of homologous alleles with and without TE insertions with epigenetic effects found no clear association between enrichment of H3K9me3 and gene downregulation [14, 16, 17]. Lee and Karpen [16] found H3K9me3 enrichment around the flanking regions of TEs in *D. melanogaster*, but this enrichment was associated with both down and upregulation of nearby genes. Huang et al. [18] extended the analysis to *D. simulans* and *D. yakuba* and concluded that TEs did not have a negative impact on nearby gene expression. Finally, Fablet et al. [19] analyzed the association of TE insertion numbers with variability in gene expression and histone enrichment. They found limited evidence for an association between H3K9me3 enrichment and TE insertion numbers, and no negative impact of TEs on the expression of nearby genes. 

While TE enrichment in H3K9me3 has been widely studied, less is known about the enrichment of active histone marks in TE sequences. Some studies, mainly using human data, have found evidence of TEs inserted in active chromatin-state regions suggesting that these TEs could be acting as *cis*-regulatory elements [20–23]. However, the majority of analyses available are based on single genomes and could not discern whether TEs induced the enrichment of active marks or were inserted into regions that were already enriched for those marks. Indeed, the few studies comparing alleles with and without TE insertions found that at least some TE families preferentially insert into genomic regions enriched for histone marks [24–26]. In *D. melanogaster*, there is evidence for an individual TE insertion that, under oxidative stress conditions, recruits an active epigenetic mark, inducing changes in the expression of adjacent genes [27]. So far, there is only one genome-wide study of TEs enriched for an active histone mark (H3K4me3) that found that the number of TE insertions was not associated with the level of this active mark in gene regions [19]. Therefore, our understanding of how TEs contribute to the genome-wide enrichment of active marks is still very limited.

Finally, whether TE insertions disrupt the epigenetic landscape of the regions where they insert has not been widely explored. There is evidence in *D. melanogaster* for the depletion of active marks in the regions flanking TE insertions [16]. Depletion of the repressive mark H3K9me2 was also found specifically for *roo* family insertions [28]. However, whether this pattern is TE-induced, and whether it impacts gene expression has not been investigated yet.

In this work, we performed a thorough study of the epigenetic effects of 2235 polymorphic TEs and their potential effects on nearby gene expression using ChIP-seq data, for a repressive (H3K9me3) and an active histone mark (the enhancer-related H3K27ac mark [29]), and transcriptomic data obtained from head, gut, and ovary of five *D. melanogaster* strains. The five strains, collected from diverse eco-geographical locations, have high-quality de novo reference genomes and TE annotations available, which allowed us to systematically characterize the epigenetic effect of TEs [30, 31]. We analyzed (i) whether TE insertions induced epigenetic changes: both enrichment and depletion of repressive and active histone marks; (ii) whether the epigenetic effect of these TEs was body part-specific; (iii) whether these effects were associated to particular TE families; and (iv) whether TEs with epigenetic effects are associated with gene expression changes and their potential functional implications.

## Results

### TEs induce the enrichment of active and repressive histone marks differentially across body parts

**Fig. 1:**
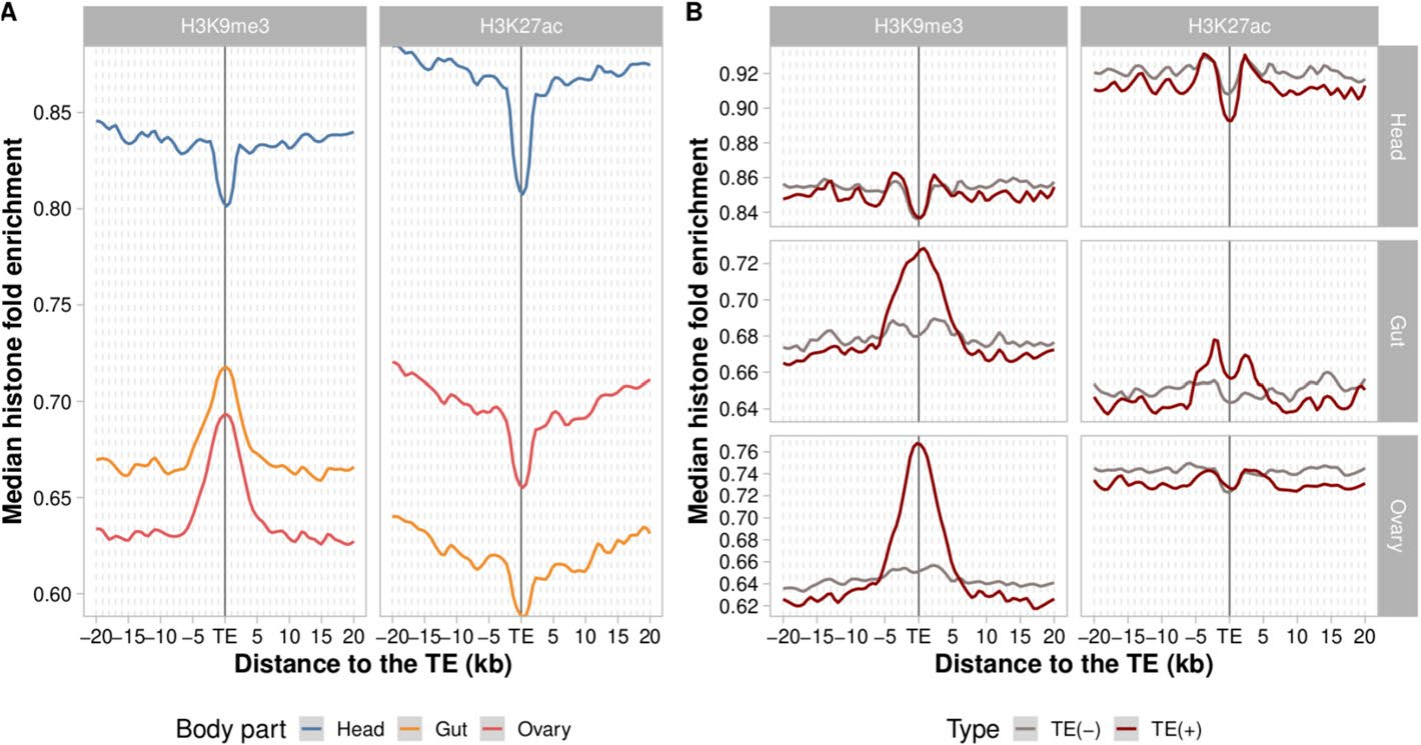
Epigenetic states in the ± 20 kb TE flanking regions across body parts. **A** Median H3K9me3 and H3K27ac fold enrichment of all TEs annotated in the five genomes analyzed (*n* = 4823 TEs). Head (blue line), gut (orange line), ovary (red line). **B** Median H3K9me3 and H2K27ac fold enrichment of polymorphic TEs (*n* = 2325 TEs). The red line represents histone mark enrichment of genomes with a TE [TE(+)] and the gray line represents histone mark enrichment of genomes without a TE [TE(−)]. The median was calculated for H3K9me3 and H3K27ac fold enrichment across all sequences flanking the analyzed TEs in the five genomes. Plots were generated using LOESS smoothing (span = 10%)

To gain insight into the epigenetic effects of TEs across adult body parts, we took advantage of the high-quality de novo TE annotations and ChIP-seq data, available for the repressive mark H3K9me3 and for the enhancer-related mark H3K27ac, from three biological replicates in the head, gut, and ovary of five *D. melanogaster* European strains [30, 31]. Four of them are inbred strains that were collected in Finland (AKA-017), Denmark (JUT-011), Germany (MUN-016), and Spain (TOM-007), and the one collected in Serbia (SLA-001) is an isofemale strain (see Methods). We first analyzed the average epigenetic states of 4823 euchromatic TE flanking sequences (± 20 kb) across the five genomes and the three body parts (see Methods). In the adult head, we observed an average depletion of the repressive mark H3K9me3 (Fig. 1A and Additional file 1: Table S1A). In the gut and ovary, there is a strong average enrichment of H3K9me3 in the TE flanking sequences, as previously described in *D. melanogaster* embryos [16] (Fig. 1A). Regarding the active mark, all three body parts showed a depletion of H3K27ac, supporting previous observations that active histone modifications are depleted near TE insertions genome-wide [16] (Fig. 1A and Additional file 1: Table S1A). However, by analyzing the average epigenetic effects of all TEs annotated in the genomes studied, we cannot discard that these patterns are due to the preferential insertion of TEs in regions enriched or depleted for active and repressive histone marks [24–26, 32].

To test whether the observed enrichment and depletion of histone marks is TE-induced, we focused on polymorphic TEs, for which we can compare the epigenetic states of the TE flanking regions in genomes with and without the TE insertions. We performed this analysis for 2325 polymorphic TEs (see Methods). In the head, we did not observe differences between the genomes with and without TEs, suggesting that TEs are not driving the previously observed depletion in the H3K9me3 mark (Fig. 1B and Additional file 1: Table S1B). On the other hand, TE insertions were associated with H3K9me3 enrichment in gut and ovary (6.4% and 16.6% enrichment in the flanking ± 1 kb window, respectively) (Fig. 1B). We hypothesized that the lower levels of H3K9me3 observed in the head could be explained by the level of expression of methyltransferases (*Su(var)3–9* and *eggless*) and demethylases (*Kdm3* and *Kdm4B*) in this tissue. We found that the expression level of *Su(var)3–9*, a key heterochromatin regulator of H3K9me3, was lower in the head compared to gut and ovary (Wilcoxon test, *p* values < 0.001 for both comparisons, Additional file 2: Fig. S1), while for *eggless*, a histone H3 N-methyltransferase, the expression was lower compared to ovary (Wilcoxon test, *p* value < 0.001) but not to gut. Indeed, the expression level of Su(var) proteins has been previously associated with the magnitude of TE’s epigenetic effects in *Drosophila* [16]. Also consistent with our hypothesis, we found that the expression level of the *Kdm3* and *Kdm4B* demethylases were higher in the head compared to gut and ovary (Wilcoxon test, *p* values < 0.05 for all comparisons, for *Kdm4B*, the comparison was only significant for head vs. gut a (Additional file 2: Fig. S1).

Regarding the active mark H3K27ac, we observed a slight depletion in genomes with TEs in head (− 1.4%), an enrichment in gut (2.3%), and no enrichment in ovary (Fig. 1B and Additional file 1: Table S1B). This contrasts with the previous observation of a general depletion of the active mark across body parts (Fig. 1A). Note that the enrichment pattern observed in gut is the one expected for active promoters, where the histone mark enrichment flanks rather than directly overlap with the enhancer sequences [33, 34] (Fig. 1B).

Although the enrichment and depletion patterns described above (Fig. 1B) were only found in the genomes that contain the TE insertions, thus suggesting that they are TE-induced, to discard that these patterns could be due to strain-specific epigenetic variation, we checked whether similar patterns were found in genomic regions that do not contain TE insertions (see Methods). We did not find consistent patterns of enrichment or depletion in the TE-free genomic regions analyzed (Additional file 2: Fig. S2) suggesting that the previously observed patterns (Fig. 1B) are most likely TE-induced.

### Individual TEs are mostly enrich for repressive marks in the ovary and for active marks in the gut and depleted similarly across body parts

While overall, polymorphic TEs induce the enrichment and depletion of repressive and active marks across body parts (Fig. 1B), the analysis of individual TE insertions allows us to gain further insight into both the percentage of enrichment and depletion and the spread of these effects to nearby regions. We considered an individual TE to have an epigenetic effect if the fold enrichment of H3K9me3, H3K27ac, or both marks (bivalent) was significantly higher or lower within ± 1 kb of the TE insertion (see Methods; [16]). For these TEs, we also computed: (i) the percentage change in enrichment or depletion compared to strains without the TE within the ± 1 kb window and (ii) the extent of spread from the TE insertion (up to ± 20 kb) with consecutive significant changes in histone marks. We computed the same metrics for our dataset of TE-free regions to show that these effects are induced by TEs and not due to epigenetic variability present in the strains (Additional file 1: Table S2A).

**Fig. 2:**
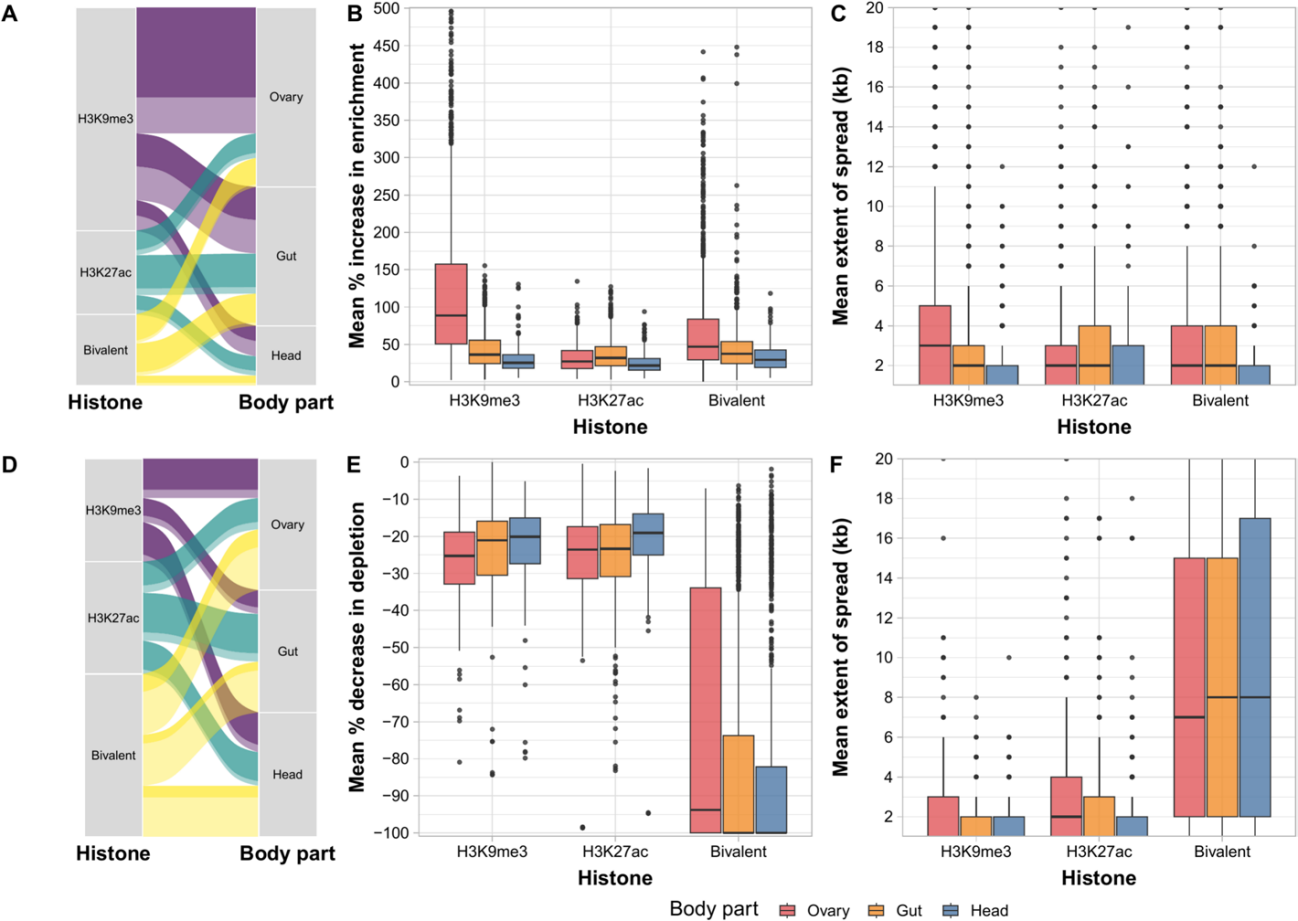
Epigenetics effects of the 1597 polymorphic TEs analyzed across body parts. **A** 1197 TEs induce the enrichment of epigenetic marks. Dark colors indicate body part-specific TEs and light colors indicate TEs that are not body part-specific. **B** Mean percentage of increase in H3K9me3, H3K27ac, and bivalent enrichment of the TEs across body parts. Outliers (TEs with epigenetic effects > 500%) are not plotted for clarity. **C** Mean extent of spread (kb) of H3K9me3, H3K27ac, and bivalent enrichment by body parts. The spread was calculated in windows of up to 20 kb adjacent to the TE insertion. **D** 552 TEs inducing the depletion of epigenetic marks. Dark colors indicate body part-specific TEs and light colors indicate TEs that are not body part-specific. **E** Mean percentage of decrease in H3K9me3, H3K27ac, and bivalent enrichment of the TEs by body parts. **F** Mean extent of spread (kb) of H3K9me3, H3K27ac, and bivalent depletion of the TEs by body parts. Boxplot colors in **B**, **C**, **E**, and **F** panels indicate the body part: ovary (red), gut (orange), and head (blue). Values and statistics related to this figure can be found in Table 1 and Additional file 1: Table S2

We found that most of the polymorphic TEs analyzed exert epigenetic effects in at least one of the body parts analyzed (71.5%, 1597/2235; Supplemental Data and Additional file 1: Table S2B, C). Among the TEs with epigenetic effects, 65.4% (1045/1597) induced the enrichment of one or both histone marks simultaneously (bivalent), 25% (400/1597) induced the depletion of one or both histone marks, and 9.6% (152/1597) induced the enrichment of histone marks in some body parts but the depletion in others. If we focus on the TEs that induce the enrichment of histone marks in at least one body part, we found that the most prevalent and strongest effect is the enrichment of H3K9me3: 816 out of 1197 TEs (Table 1 and Fig. 2A). The majority of these TEs exert their effect on the ovary (576/816), where the average percentage of enrichment and the spread of the histone mark is higher than in other body parts: 119.83% average enrichment (± 100.39) and 3.76 kb spread (± 3.5) (Wilcoxon’s test, *p* values < 0.001, Fig. 2B, C, Table 1 and Additional file 1: Table S2D). Head, in contrast, is the body part with less TEs inducing the enrichment of H3K9me3 compared to gut and ovary (140 TEs, χ^2^ test, *p* values < 0.001). Although we observed an average enrichment of H3K27ac only in the gut (Fig. 1B), we found that TEs also enrich for the active mark in other body parts (Fig. 2A). From 345 TEs that induce the enrichment of the active mark, half of them exert their effect in the gut (183/345), which is a percentage significantly higher than the TEs found in the ovary (112 TEs, χ^2^ test, *p* value < 0.001) and the head (88 TEs χ^2^ test, *p* value < 0.001) (Fig. 2A). The percentage of enrichment and spread is higher in the gut: 37.16% (± 21.97) and 2.92 kb (± 2.95), respectively (Wilcoxon’s test, *p* values < 0.05, Fig. 2B, C, Table 1 and Additional file 1: Table S2D). We found that the 183 TE insertions that enrich for H3K27ac in the gut were enriched for full-length copies (90 full-length vs. 93 fragments) compared to the TE insertions not enriched for H3K27ac in the gut (719 TEs: 264 full-length and 457 fragments; Fisher’s exact test, *p* value < 0.01), suggesting that some of these insertions might be active. We also identified 304 TEs that induced the enrichment of both marks simultaneously (bivalent) (Fig. 2A). This effect is observed in similar proportions in gut (147 TEs) and ovary (133 TEs) (χ^2^ test, *p* value = 0.29) with stronger effects in the ovary than in the gut (Wilcoxon’s test, *p* values < 0.05; Fig. 2B, C, Table 1 and Additional file 1: Table S2D).

**Table 1:** Epigenetic effects of the 1597 polymorphic TEs analyzed in this study

Effect	Histone	Body part	Number of TEs	Average spread in kb (SD)	Average enrichment in % (SD)
Enrichment	H3K9me3	–	816	3.25 (± 3.22)	86.01 (± 87.83)
	H3K27ac	–	345	2.78 (± 2.95)	36.28 (± 99.14)
	Bivalent	–	304	3.12 (± 3.41)	54.56 (± 54.7)
	H3K9me3	Gut	305	2.83 (± 2.94)	42.8 (± 24.97)
	H3K9me3	Head	140	1.88 (± 1.48)	29.15 (± 16.87)
	H3K9me3	Ovary	576	3.76 (± 3.5)	119.83 (± 100.39)
	H3K27ac	Gut	183	2.92 (± 2.95)	37.19 (± 21.98)
	H3K27ac	Head	88	2.34 (± 2.34)	36.74 (± 201.93)
	H3K27ac	Ovary	112	2.87 (± 3.34)	34.42 (± 38.8)
	Bivalent	Gut	147	3.15 (± 3.19)	44.04 (± 34.95)
	Bivalent	Head	45	1.58 (± 1.22)	33.19 (± 18.56)
	Bivalent	Ovary	133	3.59 (± 3.94)	73.23 (± 72.17)
Depletion	H3K9me3	–	200	1.92 (± 1.83)	− 24.06 (± 13.05)
	H3K27ac	–	223	2.73 (± 3.38)	− 24.21 (± 12.17)
	Bivalent	–	178	7.32 (± 6.91)	− 64.55 (± 33.13)
	H3K9me3	Gut	52	1.6 (± 1.24)	− 24.28 (± 14.13)
	H3K9me3	Head	87	1.62 (± 1.21)	− 21.77 (± 11.01)
	H3K9me3	Ovary	86	2.42 (± 2.44)	− 26.67 (± 11.54)
	H3K27ac	Gut	104	2.55 (± 2.92)	− 25.34 (± 12.73)
	H3K27ac	Head	73	2.13 (± 2.57)	− 20.63 (± 11.84)
	H3K27ac	Ovary	68	3.66 (± 4.44)	− 25.66 (± 14.13)
	Bivalent	Gut	111	7.66 (± 6.97)	− 71.04 (± 33.03)
	Bivalent	Head	111	7.95 (± 6.97)	− 73.27 (± 32.27)
	Bivalent	Ovary	133	7.81 (± 6.85)	− 63.87 (± 33.26)

In contrast to histone enrichment, which likely results from histone recruitment dependent on specific sequences within TEs, depletion is likely due to the insertional disruption of these sequences in the genome. Therefore, we hypothesized that TEs would similarly deplete H3K9me3 and H3K27ac across the three body parts. As expected, we found that TEs depleted H3K9me3 and H3K27ac in similar proportions (χ^2^ test, *p* value = 0.17; Fig. 2D, Table 1 and Additional file 1: Table S2B, C) and that histone mark depletion was similar across body parts (χ^2^ tests, *p* values > 0.1, Table 1 and Additional file 1: Table S2E). Notably, the bivalent depletion is the one with the highest effects and longest spread: 9.13 kb (± 7.05) and − 78.1% (± 31.23), respectively (Wilcoxon’s test, *p* values < 0.001; Fig. 2E, F and Additional file 1: Table S2D).

Finally, we found that TEs inducing the enrichment and depletion of epigenetic marks are highly body part-specific: 757 out of the 1197 TEs that enrich and 376 out of 552 TEs that deplete exert their epigenetic effect in only one body part (Fig. 2A and D and Additional file 1: Table S2B). Focusing on TEs that induce histone mark enrichment, the most body part-specific effects were the enrichment of H3K27ac and bivalent marks (308/345 and 285/304, respectively). In the case of TEs inducing depletion, the most body part-specific TEs are those depleting for H3K27ac and H3K9me3 (177/200 and 201/223, respectively) (Fig. 2D and Additional file 1: Table S2B).

Overall, the majority of individual TE insertions induced the enrichment of H3K9me3 in the ovary and the enrichment of H3K27ac in the gut, although we also found enrichment of these histone marks in other body parts. Moreover, the epigenetic effects of TEs are not restricted to the enrichment of histone marks, as we also identified 552 TEs that depleted histone marks.

### TE families differed in the proportion of copies enriched for H3K9me3 and H3K27ac

**Fig. 3:**
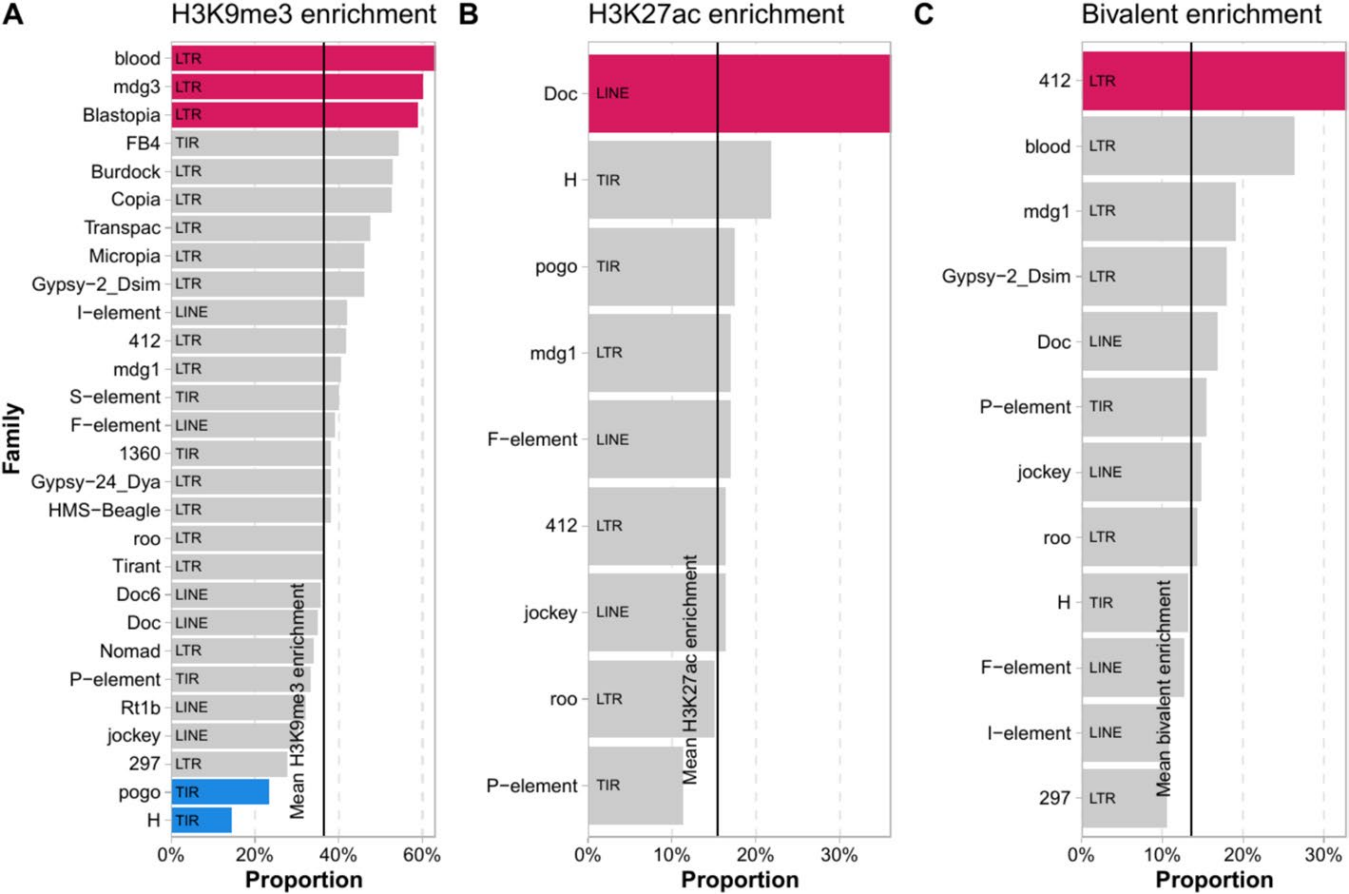
Proportion of TEs per family enriched for epigenetic marks. **A** Proportion of copies per family that are enriched for H3K9me3 epigenetic mark. **B** Proportion of copies per family that are enriched for H3K27ac epigenetic mark. **C** Proportion of copies per family enriched for bivalent epigenetic histone marks. Red: larger proportion of copies associated with enrichment of histone mark than expected, blue: fewer proportion of copies associated with enrichment of histone mark than expected, gray: non-significant. Only families with a minimum of 8 copies with epigenetic effects and more than 20 copies in the genome were considered. Numbers in Additional file 1: Table S3

TE families vary in many properties, including transposition mechanisms, lengths, genome distribution, density, and the targeting by small RNAs [35–38]. Variation in the proportion of TEs with epigenetic effects, spread, and enrichment of H3K9me3 has already been reported across families [16]. We thus tested whether we could find variation in the epigenetic effects of TEs depending on their family identity. We focused on families with > 20 genome copies, and we found that TE families that showed a larger proportion of copies associated with the enrichment of H3K9me3 and with the enrichment of the two marks were all LTRs: *blood*, *mdg3*, *Blastopia*, and *412* (Fig. 3A and C and Additional file 1: Table S3A). *Pogo* and *H* elements were the only families with significantly fewer proportion of insertions associated with H3K9me3 enrichment (Fig. 3A). On the other hand, a LINE family, *Doc*, was the only family that showed a significantly larger proportion of copies enriched for H3K27ac (Fig. 3B). In the case of TEs associated with histone mark depletion, we do not expect them to be enriched for particular families as the effect is not dependent on the TE itself but rather on whether the TE inserts in a sequence that recruits chromatin modifiers. Indeed, we only found one family with a larger proportion of copies depleted for H3K27ac: *FB4* (Additional file 1: Table S3B).

### Sixty percent of the TEs that induce epigenetic changes have an effect on the expression of adjacent genes

**Fig. 4:**
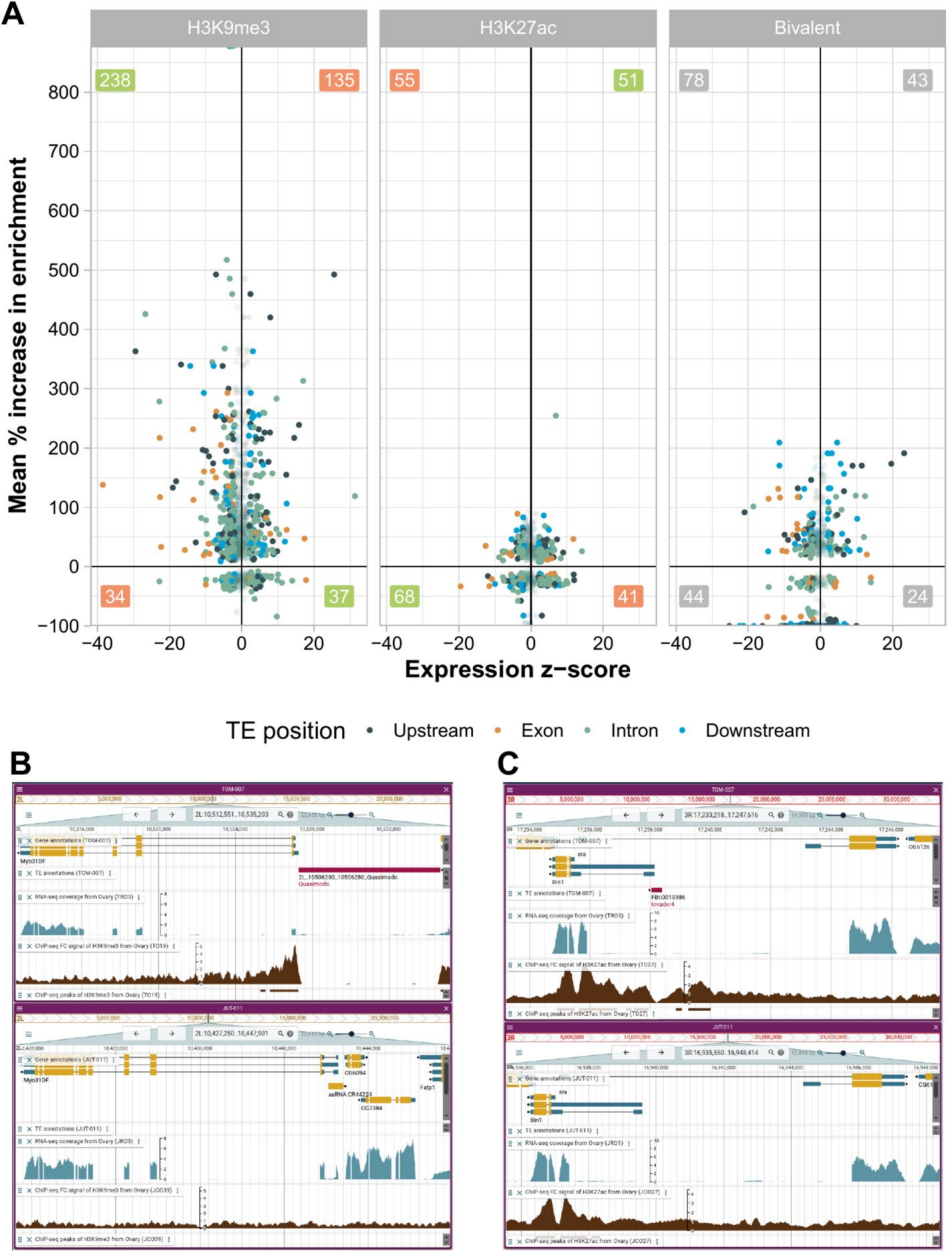
TEs with epigenetic effects are associated with gene expression changes. **A** H3K9me3, H3K27ac, and bivalent enrichment and expression *z*-score for genes with and without nearby TE insertions. The *z*-score for gene (TE +) vs. gene (TE −) expression (x-axis) and mean percentage of increase of the histone mark enrichment (y-axis) is plotted for TE-gene pairs. Green numbers indicate TE-gene pairs with the expected association between the epigenetic mark and the expression level, red numbers indicate TEs when the association is the opposite, and gray when there are no expectations. **B** Snapshot from DrosOmics browser for a TE that is enriched for H3K9me3 and associated with gene downregulation [30]. The *Myo31DF* gene region in two strains is shown: TOM-007 in the top (with a *Quasimodo* TE insertion in the promoter region of the gene, in purple) and JUT-11 in the bottom (without the TE insertion). There is an enrichment of H3K9me3 in the flanking regions of the insertion that is not observed when the TE is absent, and the level of expression of *Myo31DF* is lower in TOM-007 (with the insertion). The brown track represents the ChIP-seq fold-change signal of H3K9me3, and the turquoise track the RNA-seq coverage levels. **C** Snapshot from DrosOmics browser for a TE enriched in H3K27ac and associated with gene upregulation [30]. The *Bin1* and *sra* gene regions in two strains are shown: TOM-007 in the top (with an *Invader4* TE insertion in the promoter region of *Bin1* and within the first intron of *sra*) and JUT-011 in the bottom (without the TE insertion). There is a H3K27ac peak flanking the TE that is not present when the TE is absent, and the level of expression of *Bin1* is higher in the genome of TOM-007 (with the insertion). As **B**, the brown track represents the ChIP-seq fold-change signal of H3K27ac and the turquoise track the RNA-seq coverage levels. Statistical analyses and expression data are included in Additional file 1: Table S4A

To assess whether the epigenetic effect of TEs has an impact on the expression of nearby genes, we analyzed matching RNA-seq data available for all the strains and body parts [30]. Out of the 1597 TEs that have epigenetic effects, 1168 TEs had an expressed gene located within the spread of the histone mark enrichment/depletion. We calculated the *z*-score of the gene expression change between strains with and without the TE insertion. A negative *z*-score indicates that the expression level of the gene adjacent to the TE is lower than the gene expression of the same gene when the TE is not present (see Methods). We found that 703 TEs (60%) have a significant impact on gene expression (728 genes): 389 TEs were associated with reduced gene expression, 231 with increased gene expression, and 83 were associated with increased gene expression in one body part and reduced expression in another (Table 2). We found that 70% (238/340) of the TEs that are enriched for the repressive mark, and associated with gene expression changes, are indeed associated with the downregulation of the nearby gene (Fig. 4A, B, Table 2 and Additional file 1: Table S4A). A permutation test showed that while for some strains and tissues, the association of a TE with the downregulation of the nearby gene is likely due to the presence of the repressive histone marks, in other cases we cannot discard that the effect on expression is due to the presence of the TE itself (Additional file 1: Table S4B). We further tested whether the effect on gene downregulation of TEs enriched for the repressive mark is significant compared with TE-free regions that are also enriched for H3K9me3. We found that this was the case for the majority of strains and body parts (Additional file 1: Table S4C).

**Table 2:** TEs with epigenetic effects associated with gene expression changes. FC: fold change, of the difference in expression of genes in strains with and without the TE insertion

Effect	Histone mark	TEs	TEs inducing a significant change in expression	TEs that induce downregulation (range log_2_FC and mean)	TEs that induce upregulation (range log_2_FC and mean)
Enrichment	H3K9me3	606	340	238 (− 0.08 to − 79.7, − 1.5)	135 (0.08–13.7, 1.5)
	H3K27ac	244	102	55 (− 0.14 to − 79.72, − 2.46)	51 (0.12–5.7, 1.2)
	Bivalent	216	112	78 (− 0.14 to − 79.72, − 2.1)	43 (0.078–13.7, 1.6)
Depletion	H3K9me3	149	70	34 (− 0.12 to − 79.72, − 3.23)	37 (0.11–2.2, 0.79)
	H3K27ac	171	101	68 (− 0.081 to − 79.72, − 2.2)	41 (0.13–5.8, 1.1)
	Bivalent	106	59	44 (− 0.1 to − 79.72, − 2.1)	24 (0.18–2.9, 1.1)

On the other hand, 50% (51/102) of the TEs that induced the enrichment of the active mark are associated with gene upregulation (Fig. 4A and C, Table 2 and Additional file 1: Table S4A). Overall, the effect of TEs enriched for active histone marks on gene upregulation is not significant compared with TE-free regions enriched for the same active histone mark (Additional file 1: Table S4C). Finally, the depletion of the repressive mark was associated with gene upregulation in 52% of the TEs (37/70) while the depletion of the active mark with gene downregulation in 67% of the TEs (68/101). Regarding TEs that either induce bivalent enrichment or depletion, they are mostly associated with gene downregulation (70% 78/112, and 75% 44/59, respectively).

The position of the TE relative to the gene (upstream, downstream, UTRs, CDS, or intron) was not associated with the direction of the expression change observed for H3K9me3 or H3K27ac enrichment and depletion (Additional file 1: Table S4D). Head and ovary were the body parts with more genes being significantly up or downregulated due to the epigenetic effects of TEs compared to gut (head: 47% 228/482, gut: 41% 305/746, and ovary: 49% 427/879, χ^2^ test *p* value = 0.031 and 0.002, respectively).

Finally, we did not find differences on the histone mark spread, percentage of enrichment, TE location regarding the nearby gene (upstream, downstream, exonic, and intronic), TE length distribution, and TE frequencies, between TEs with epigenetic effects associated with significant changes in expression and TEs with epigenetic effects that do not affect expression (Additional file 1: Table S4E). This result suggests that the epigenetic effect of a TE, or lack thereof, is not explained only by the TE itself but also depends on the genomic context where it is inserted [15, 18].

### Twelve of the TEs affecting gene expression through epigenetic changes are likely to be adaptive

It has been hypothesized that the epigenetic silencing of TEs could have a deleterious effect through the downregulation of nearby genes [14]. Indeed, we found that TEs that are enriched for the repressive histone mark and are associated with gene downregulation are enriched for low frequent insertions, as expected if these TEs have deleterious effects [16] (Fisher’s exact test, *p* value < 0.001, Additional file 1: Table S4F). Note that the low frequency of these insertions could also be indicative of recent TE activity [39]. On the other hand, nine of these TEs are present at high frequencies (> 10%) suggesting that they might be adaptive, with one of these TEs present at a higher frequency compared to other insertions from the same family and another two TEs previously identified as candidate adaptive TEs (Additional file 1: Table S4G). These nine TEs are located nearby 11 genes related to *transport/localization* (5/11), *cell organization/biogenesis* (4/11), and *cell cycle proliferation* (3/11) (Additional file 1: Table S4G). Additionally, one TE (*2R_14883304_14883308_BS2*) is associated with three cytochrome P450 genes (*Cyp6a20*, *Cyp6a21*, and *Cyp6a9*) which are associated to insecticide resistance in *Drosophila* [40].

Two of the 51 TEs that are enriched for the active histone mark and associated with gene upregulation are also present at high frequencies (> 10%; Additional file 1: Table S4G). The three genes associated with gene upregulation, *Bin1*, *sra*, and *Svil*, are all related to *cell organization/biogenesis*. One TE (*FBti0019386*) affecting both *Bin1* and *sra* was already described to be associated with gene upregulation in cold-stress conditions and in response to infection in *D. melanogaster* (Fig. 4C, [41, 42]). The other TE, associated with *Svil* upregulation, has also been previously identified as a candidate adaptive TE (Additional file 1: Table S4G).

If we focus on TEs inducing depletion of histone marks, for the 37 TEs depleting H3K9me3 that induce gene upregulation, seven are present at high frequencies (> 10%), with one TE showing a significantly higher frequency compared with all other insertions in that family. Five of these seven TEs are located nearby genes enriched for *cell organization/biogenesis* processes and four have previous evidence for an adaptive role (Additional file 1: Table S4G). *FBti0019170*, affecting the *kuzbanian* gene, has already been associated with adaptation in response to zinc-stress tolerance [43, 44] (Additional file 1: Table S4G). While *FBti0019386* was associated with H3K27ac in the gut in two of the strains analyzed, we found that this TE is also associated with H3K9me3 depletion in the gut in another strain, further suggesting that the epigenetic effect of TEs is context dependent.

Among the 68 TEs inducing the depletion of the active mark associated with gene downregulation, there are 6 TEs present at high frequencies (> 10%), associated with only two genes with a known function: one gene is a small nuclear RNA and the other one is a rho GTPase activating protein (Additional file 1: Table S4G). Three of these TEs were previously identified as candidate adaptive TEs (Additional file 1: Table S4G).

Overall, we found that 12 of the TEs with epigenetic effects and associated with gene expression changes are present at higher population frequencies compared to other insertions in the same family, suggesting that they have increased in frequency due to positive selection rather than genetic drift, or have previous evidence for an adaptive role (Additional file 1: Table S4G).

### Genes regulated by TEs with epigenetic effects in head have functions associated with catabolic process and signal transduction

To analyze whether TEs with epigenetic effects are regulating genes with similar functions, potentially indicative of a rewiring of specific gene regulatory networks, we performed a Gene Ontology (GO) enrichment analysis [45]. We found that genes upregulated in the head were enriched for catabolic processes and signal transduction biological processes (e.g., *negative regulation of cell communication, response to stimulus, and signaling*; Additional file 1: Table S5). In the gut and in the ovary, the latter being the body part with more genes associated with TEs, genes located nearby TEs with epigenetic effects were not enriched for any GO terms (Additional file 1: Table S5).

## Discussion

In this work, we have assessed the epigenetic effects of 2235 polymorphic TEs across three *D. melanogaster* adult body parts (Fig. 1B). The availability of high-quality TE genome annotations and ChIP-seq data for three body parts and five genomes has allowed us to determine that 1197 of the TEs analyzed induced the enrichment of repressive or active histone marks (or both) rather than being inserted in regions already enriched for these marks (Fig. 2 and Additional file 2: Fig. S2). Including RNA-seq data in the analysis has also allowed us to investigate the functional consequences of TE insertions on gene expression. In contrast to previous studies [6, 15–19], we have found evidence for an effect of TEs on neighboring gene expression. Overall, we observed that 70% (238/340) of the TEs that were enriched for H3K9me3, and associated with gene expression changes, lead to the downregulation of the genes located within the spread of this histone mark (Fig. 4A and Table 2). Previous evidence for an association between H3K9me3 enrichment and gene downregulation in *D. melanogaster* was limited [reviewed in 1,14]. These studies either restricted their analyses to reference TE insertions, annotated in a single genome [15, 28], or used RNA-seq and ChIP-seq data generated for whole animals reflecting the average epigenetic states of multiple cell types [16]. In contrast, we have analyzed de novo TE annotations in five genomes, together with ChIP-seq and RNA-seq obtained from three adult body parts of the same individuals, which allows to establish a significant association between TEs enriched for repressive histone mark and downregulation of nearby genes. Our results highlight the importance of multi-omic integrative approaches analyzing several reference genomes to get a more comprehensive understanding of the functional consequences of TEs [31].

Although smaller, a substantial proportion of the TEs enriched for H3K27ac that affect nearby gene expression were associated with the expected gene expression change: gene upregulation (51/102). While the enrichment of H3K9me3 occurs through small-RNA mediated mechanisms [5–8], the capacity of TEs to enrich for active marks has been suggested to be associated with the recruitment of chromatin modifiers by TEs [23].

Furthermore, we also showed that the epigenetic effects of TEs extend beyond histone mark enrichment as 552 of the analyzed TEs induce the depletion of histone marks in at least one body part. The percentage of TEs disrupting H3K9me3 and H3K27ac marks and the number of TEs depleting these histone marks across body parts was similar (Table 1). This pattern is not unexpected as the disruption of histone marks by TEs is due to the insertion of these sequences and not to host control mechanisms (see below). TEs disrupting epigenetic marks were also associated with gene expression changes. We found that 67% of the TEs inducing the depletion of the active mark were associated with gene downregulation, and that 52% associated with the depletion of H3K9me3 were associated with gene upregulation. Overall, both for TEs that enrich and for TEs that deplete histone marks, the percentage of TEs associated with the expected change of expression was high (Fig. 4A). While some TEs were associated with the opposite pattern of expression, it is known that the association between epigenetic marks and gene expression is complex (e.g., [46, 47]). Indeed, previous evidence for a correlation between H3K27ac enrichment and gene upregulation is weak [48, 49], which is consistent with results suggesting that besides H3K27ac enrichment enhancer activity requires additional acetylation events on other histone lysine residues [50].

By analyzing expression data on three distinct body parts—head, gut, and ovary—we found that the epigenetic effects of TEs are body part-dependent. Overall, we have observed that the most prevalent epigenetic effect is the enrichment of H3K9me3 in ovaries (576 TEs), driven by the piRNA pathway [51]. This is not unexpected, as TE insertions in the female germ line can compromise the genome integrity and the fitness of the offspring [8, 52, 53]. On the contrary, the head body part exhibited fewer TEs enriched for repressive marks (140 TEs), which could be explained by the lower expression of *Su(var)3–9* gene, which encodes a key heterochromatin regulator of H3K9me3 (Additional file 2: Fig. S1). The lower enrichment of TEs for H3K9me3 could be linked to a potentially higher rate of TE mobilization in the brain, as has been observed for *Drosophila* neurons [54] and in the human brain ([55–57]; but see also [58, 59]). Thus, while our data suggest a lower repression of TEs in the head, more research is needed to elucidate whether this leads to an increased mobilization of TEs in the brain. We have also found that in the gut, the prevalent epigenetic effect of TEs is the enrichment of the active mark H3K27ac. TEs have previously been reported to be enriched in transcriptionally active chromatin (rich in acetylated histone H3) in the *Drosophila* midgut [60]. Indeed, 5 of the 41 families enriched for H3K27ac in the gut were also found to be active by Siudeja et al. [60]. During viral infections, the reverse transcriptase of certain TEs is used to generate copies of the invading virus, amplifying the immune response against viral infections [61, 62]. A higher expression of TEs in the gut, induced by H3K27ac, might thus be seen as a trade-off between TE activity and response to viral infections occurring in the gut [61].

## Conclusions

Overall, our study provides evidence that the epigenetic effect of TEs goes beyond gene silencing and shows that TEs can also affect gene expression by disrupting the epigenetic marks of the regions where they insert. In the ovary, TEs are primarily enriched for the repressive H3K9me3 mark, probably reflecting the need to protect the germ line, while in the head, TEs affect genes involved in specific biological functions, suggesting a potential role in rewiring gene regulatory networks. By comparing several TE-related metrics between TEs with epigenetic effects that do and do not affect gene expression (i.e., histone mark spread, percentage of enrichment, TE position, TE length distribution, and TE frequencies), we concluded that the epigenetic consequences of TE insertions are genomic context dependent. Furthermore, 12 TEs with epigenetic effects that were associated with a change in gene expression were present at significantly higher population frequencies or were previously linked to an adaptive phenotypic effect or reported in genome-wide screenings for adaptive signals (Additional file 1: Table S4G). Because the majority of TEs inducing epigenetic changes were found at low population frequencies, the analysis of more genomes, and thus of an even more complete set of TEs present in natural populations, should help elucidate the overall role of TEs in generating gene regulatory novelty by inducing or disrupting epigenetic states.

## Methods

### Strain genomes and TE annotations

We have used available de novo reference genomes of five European *D. melanogaster* natural strains: AKA-017, JUT-011, MUN-016, SLA-001, and TOM-007 [31]. For these genomes, there are high-quality annotations of transposable elements [31]. In brief, we analyzed the 4823 TEs annotated in euchromatic region that are non-nested (Additional file 1: Table S6). Genomes and TE annotations are available for visualization at the DrosOmics genome browser [30].

### TE breakpoints calls

To determine the TE insertion location (breakpoint) in genomes lacking a TE insertion, we focused on polymorphic TEs, a total of 3878 insertions. We extracted the ± 500 bp flanking regions of each TE using *BEDOPS* (v2.4.39, [63]) and *BEDtools* (v2.30.0, [64]) and used *minimap2* (with parameters *-ax splice -G100k -B2 -t 4 -C5*) (v2.17, [65]) to map such flanking regions in the genome lacking the TE. We called a TE breakpoint location when the mapping of the upstream, downstream, and concatenated flanking region was unique, allowing a gap/overlap of ± 50 bp between the upstream and downstream regions. We could determine the insertion location for 2325 (60%) of the TEs analyzed. The ± 20 kb flanking region of each TE insertion was divided into 20 non-overlapping 1 kb windows respectively to perform ChIP-seq enrichment analyses.

### RNA-seq and ChIP-seq data for H3K9me3 and H3K27ac in three body parts

RNA-seq data and ChIP-seq data for H3K9me3 and H3K27ac in three body parts for five strains were obtained from Coronado-Zamora et al. [30], where a full description of the protocols used to generate these data can be obtained. Briefly, head, gut, and ovary body parts of each strain were dissected at the same time from the same individual flies. For each body part and strain, three replicates of 30 females aged 4–6 days were processed. RNA-seq library preparation was performed using the TruSeq Stranded mRNA sample prep kit from Illumina and sequenced using Illumina 125 bp paired-end reads. ChIP-seq libraries were performed using TruSeq ChIP library preparation kit. Sequencing was carried out in an Illumina HiSeq 2500 platform, producing 50 bp single-end reads. Raw RNA-seq and ChIP-seq data are available in the NCBI Sequence Read Archive (SRA) database (BioProject PRJNA643665, [66]) and can be retrieved and visualized through DrosOmics genome browser [30].

### ChIP-seq data processing

ChIP-seq reads were processed using *fastp* (v.0.20.1, [67]) to remove adaptors and low-quality sequences. Processed reads were mapped to the corresponding de novo reference genome using the *readAllocate* function (*chipThres* = *500*) of the *Perm-seq* R package (v0.3.0, [68]), with *Bowtie* (v1.2.2, [69]) as the aligner and the Csem program (v2.3, [70]) in order to try to define a single location to multi-mapping reads. In all cases, *Bowtie* was performed with default parameters selected by *Perm-seq*.

Then, we used the ENCODE ChIP-Seq caper pipeline (v2, [71]) in histone mode, using *Bowtie 2* as the aligner [69], disabling pseudo replicate generation and all related analyses *(chip.true_rep_only* = *TRUE*) and pooling controls (i.e., ChIP-seq input, *chip.always_use_pooled_ctl* = *TRUE*). The MACS2 peak caller was used with default settings [72]. The signal value tracks produced by MACS2 were processed as follows. We used *bigWigToBedGraph* from UCSC (available at https://genome.ucsc.edu/goldenPath/help/bigWig.html) to transform bigWig files to BED files. Then, for each replicate, we used *bedmap* with the *mean* option (v2.4.39, [63]) to obtain the average signal values in 10 bp windows. Finally, the three replicates were averaged using *bedmap mean* to obtain a single track value for each sample that was used to estimate the spread and enrichment of histone marks adjacent to TE insertions. The enrichment signal by MACS for the two histone marks in each body part and strain is shown in Additional file 2: Fig. S3. As expected, H3K9me3 is enriched in heterochromatic regions (near pericentromeric regions), while H3K27ac is enriched in euchromatic regions and depleted in pericentromeric regions. However, two strains (AKA-017 and JUT-011) showed a more noisy H3K9me3 coverage and slightly different H3K27ac coverage in the head body part. To ensure that enrichment patterns were not affected by these two strains, we repeated Fig. 1A excluding these two strains. The overall patterns remained consistent (Additional file 2: Fig. S4). Finally, to assess heterogeneity across strains, the same figure (Fig. 1A) was plotted for individual strains. Overall, the patterns are consistent across strains, with H3K27ac showing depletion across the three body parts and H3K9me3 showing enrichment in the gut and ovary, while both marks are depleted in the head (Additional file 2: Fig. S5).

### ChIP-seq data analysis

From the 2325 polymorphic TEs, we computed two statistics when comparing pairs of TE(+) and TE(−) genomes [16]: the *percentage of increase or depletion of H3K9me3/H3K27ac enrichment* and the *extent of H3K9me3/H3K27ac spread*. We used Wilcoxon’s test (with the *wilcox.test* function from the *stats* package in R, v3.6.3, [73]) to assess if the H3K9me3/H3K27ac enrichment or depletion in the *i*th upstream and downstream windows differs significantly between pairs of TE(+) vs. TE(−) genomes. The most distant windows considered are 20 kb from TE insertions. The *percentage increase or depletion of H3K9me3/H3K27ac enrichment* is the difference of median H3K9me3 enrichment between two genomes in the ± 1 kb windows immediately next to the TE insertion, divided by the enrichment level for the strain without TE. The *extent of H3K9me3/H3K27ac spread* is the farthest window in which the H3K9me3/H3K27ac enrichment or depletion is consecutively and significantly higher or lower in the genome with TE compared with the genome without the TE. When the farthest windows are different between the left and right sides of a TE insertion, we used the window closer to the TE for the *extent of H3K9me3/H3K27ac spread* (to be conservative). To consider a TE for further analyses, we allowed only one different comparison across pairs of TE(+)-TE(−) genomes (that could be due to inconsistencies in the epigenetic change, no statistical enrichment or NA data, Additional file 2: Fig. S6). We excluded 49 TEs that did not fulfill the criteria in any of the body parts. From the remaining 2276 TEs, 1597 have epigenetic effects in at least one body part and at least for one histone mark. Six hundred thirty-eight TEs do not have an effect (no enrichment) in any of the body parts and 41 have a “mix” effect (i.e., enrich for one mark and deplete for the other) and were not analyzed. Therefore, the final dataset analyzed was of 2235 TEs (Additional file 2: Fig. S7).

### Analysis of histone enrichment patterns in TE-free regions

For every strain-specific TE insertion (2171 TEs), we selected an euchromatic region without a TE insertion annotated within 1 kb. Note that some of the TE insertions have more than one body part-dependent effect or affect more than one gene, we thus selected as many TE-free regions as TE-associated regions affected. In total, we analyzed 5088 TE-free regions. For these 5088 TE-free regions, we plotted the median H3K9me3 and H3K27ac in ± 20 kb regions across body parts and genomes.

### RNA-seq data processing to obtain gene expression levels

To obtain gene expression levels from RNA-seq, we used a reference-guided transcriptome assembly obtained for each strain following Pertea et al. [74]. A detailed description can be found in Coronado-Zamora et al. [30]. To obtain the gene count estimations normalized for TMM (trimmed mean of the M-values), we first used the *prepDE.py* script from StringTie [75] to obtain a matrix of gene counts, with parameter *-l 125*. Next, we used the *edgeR* R package (v3.28.0, [76]) to create a *DGEList* object from the table of counts using the *DGEList* function. Next, we used *calcNormFactors* function with the TMM method to calculate the normalization factors to scale the raw library sizes. Finally, we used the function *cpm* to obtain the normalized counts using the TMM normalization factors.

### *Z*-score calculation

We assigned each of the TEs with epigenetic effect to the gene(s) overlapping or adjacent, taking into account the spread of the histone mark. For each pair of TE-gene, a *z*-score was calculated as the mean of gene expression of gene with TE (averaging the three available replicates) minus the mean expression of gene without the TE, divided by the standard error of both groups of genes. A negative *z*-score indicates that the gene associated with the TE has lower expression compared to the gene without a TE insertion, while a positive *z*-score indicates that the gene associated with the TE has a higher expression.

### TE positional effect vs. TE insertion with epigenetic effects on gene expression

To determine whether TEs have an impact on gene expression because of their epigenetic effects and not by their presence alone (positional effect), we analyzed all strain-specific TE insertions located within 1 kb of genes. Using a permutation test (sampling = 1000), we compared the observed effects of TEs with epigenetic effects and associated with gene downregulation to random sets of TEs of equal size drawn from all the insertions annotated in the genome and matched by their location relative to genes (intron, 5′/3′ UTRs, CDS, and upstream/downstream regions). This approach allowed us to assess whether expression changes linked to TEs with epigenetic effects are significantly greater than would be expected from the presence of the insertion alone (Additional file 1: Table S4B).

### Epigenetic effects on gene expression in TE-free vs. TE-associated regions

To evaluate whether TEs with epigenetic effects have a stronger influence on gene expression than TE-free random genomic regions, we first identified TE-free regions located nearby genes (within 1 kb) that did not overlap any annotated TE (with a minimum distance of 1 kb from any TE). These regions were selected to match the genomic contexts where TEs were previously found to exert epigenetic effects (introns, 5′ and 3′ UTRs, CDS, and upstream/downstream regions, Additional file 1: Table S4C). For each genome strain, we selected as many TE-free null regions as possible within each category. For each body part and histone mark analyzed, we focused on TE-free regions enriched for that mark in one strain relative to the others, and recorded the number of associated genes showing up or downregulation. We then conducted permutation tests (sampling = 1000) to determine whether the number of differentially expressed genes in these TE-free regions differed significantly from the number observed for TE-associated regions (Additional file 1: Table S4C).

### Gene ontology (GO) enrichment analysis

To perform the GO enrichment analysis, we used the software GOWINDA (v.1.12, [45]) using the “gene” mode and the “updownstream20000” gene-definition and only considering the genes associated with TEs with epigenetic effects (parameters: –simulations 100,000, –min-genes 2, –min-significance 1). We applied a FDR < 0.05.

### Analysis of the proportion of TE copies with epigenetic effects by family

For the TE family analysis, we considered TE families with a minimum of 20 copies in the genome and with at least 8 copies with epigenetic effects to perform the statistical analysis. We considered a TE family as significant when the number of copies exhibiting epigenetic effects was significantly higher than the respective average. Specifically, we used the averages of 36.5% (816/2235), 15.4% (345/2235), and 13.6% (304/2235) as the thresholds for H3K9me3, H3K27ac, and bivalent enrichment, respectively. To test the significance of the enrichment, we applied a χ^2^ test (using the *chisq.test* function from the *stats* package in R (v3.6.3, [73]). 

## Supplementary Information

Additional file 1: Supplementary tables.

Additional file 2: Supplementary table legends and supplementary figures.

## Data Availability

All scripts and R Markdown documents to reproduce analyses and figures have been deposited in GitHub: https://github.com/GonzalezLab/epigenetic-effects-transposons-dmelanogaster [77] and are also available in Zenodo (10.5281/zenodo.15876976). Raw RNA-seq and ChIP-seq data for five D. melanogaster strains (AKA-017, JUT-011, MUN-016, SLA-001 and TOM-007, [31]) are available in the NCBI Sequence Read Archive (SRA) database (BioProject PRJNA643665) [66].

## Declarations

### Peer review information

Tim Sands was the primary editor of this article and managed its editorial process and peer review in collaboration with the rest of the editorial team. The peer-review history is available in the online version of this article.
