# Supplementary material for: The epigenetics effects of transposable elements are genomic context dependent and not restricted to gene silencing in Drosophila
Source: Genome Biol. 2025 Aug 18;26:251. doi: 10.1186/s13059-025-03705-4 (PMC12360000; doi:10.1186/s13059-025-03705-4)
Supplement: Supplementary file 2 — Additional file 2: Supplementary table legends and supplementary figures. [file 13059_2025_3705_MOESM2_ESM.docx]

Supplemental information for:

**The epigenetics effects of transposable elements are genomic context dependent and not restricted to gene silencing in Drosophila**

Marta Coronado-Zamora and Josefa González*

Institut Botànic de Barcelona, CSIC, CMCNB, Barcelona, Spain.

*Corresponding author. E-mail: [josefa.gonzalez@csic.es](mailto:josefa.gonzalez@csic.es)

Contributing author: [marta.coronado@csic.es](mailto:marta.coronado@csic.es)

# CONTENTS

**Supplementary tables legends** page 2

**Supplementary figures** page 3

## SUPPLEMENTARY TABLES LEGENDS

**Table S1.** Average epigenetic states of TEs flanking sequences (±20 kb) across three body parts. A. Median H3K9me3 and H3K27ac fold enrichment for 4,823 TEs. B. Median H3K9me3 and H3K9me3 fold enrichment for 2,325 TEs.

**Table S2.** Epigenetic effects of 5,088 null regions and the 1,597 TEs across three body parts. A. Null regions analysis. Wilcoxon’s test comparing the spread and average enrichment of null regions vs TEs with epigenetic effects. B. Number of TEs with epigenetics effects divided by type of effect (enrichment and depletion) and number of body parts. C. Body-part specific and shared TEs separated (numbers of Figure 2A). D Wilcoxon's test results comparing enrichment and spread of histone marks. E. χ² test for TEs inducing bivalent depletion (552) across body parts.

**Table S3.** TE families enrichment. A. Enrichment in TEs that enrich for H3K9me3, H3K27ac or both (bivalent). B. Enrichment in TEs that deplete for H3K9me3, H3K27ac or both (bivalent). P-values obtained with a χ² test comparing the total number of TEs of each families with those associated to enrichment of an epigenetic mark. P-values were corrected by FDR (q-value).

**Table S4.** Gene expression analysis for TEs with epigenetic effects. A. Z-score of gene expression for each TE with epigenetic effects. Information of the TE frequency from Rech et al. 2022. B. Positional effects of TEs on gene expression. Considering strain-specific TEs and located 1kb to genes. C. TE-free regions analysis and permutation test results (n sampling = 1000). D. Post-hoc chi square comparing TE position for genes that exhibit different directions of expression. E. Wilcoxon's test comparing TE properties in TE that induce significant changes in expression vs genes that do not. Post-hoc chi square results comparing TE position. F. Fisher's exact test for TE frequency in TEs that induce H3K9me3 and H3K27ac. G. Genes associated with TE with epigenetic effects with high frequency.

**Table S5.** Gene Ontology results performed with GOWINDA. FDR < 0.05. The analysis was performed only considering genes that have a difference in expression due to a TE with epigenetic effects located in a maximum of 20kb.

**Table S6.** Number of TEs analyzed.

## SUPPLEMENTARY FIGURES


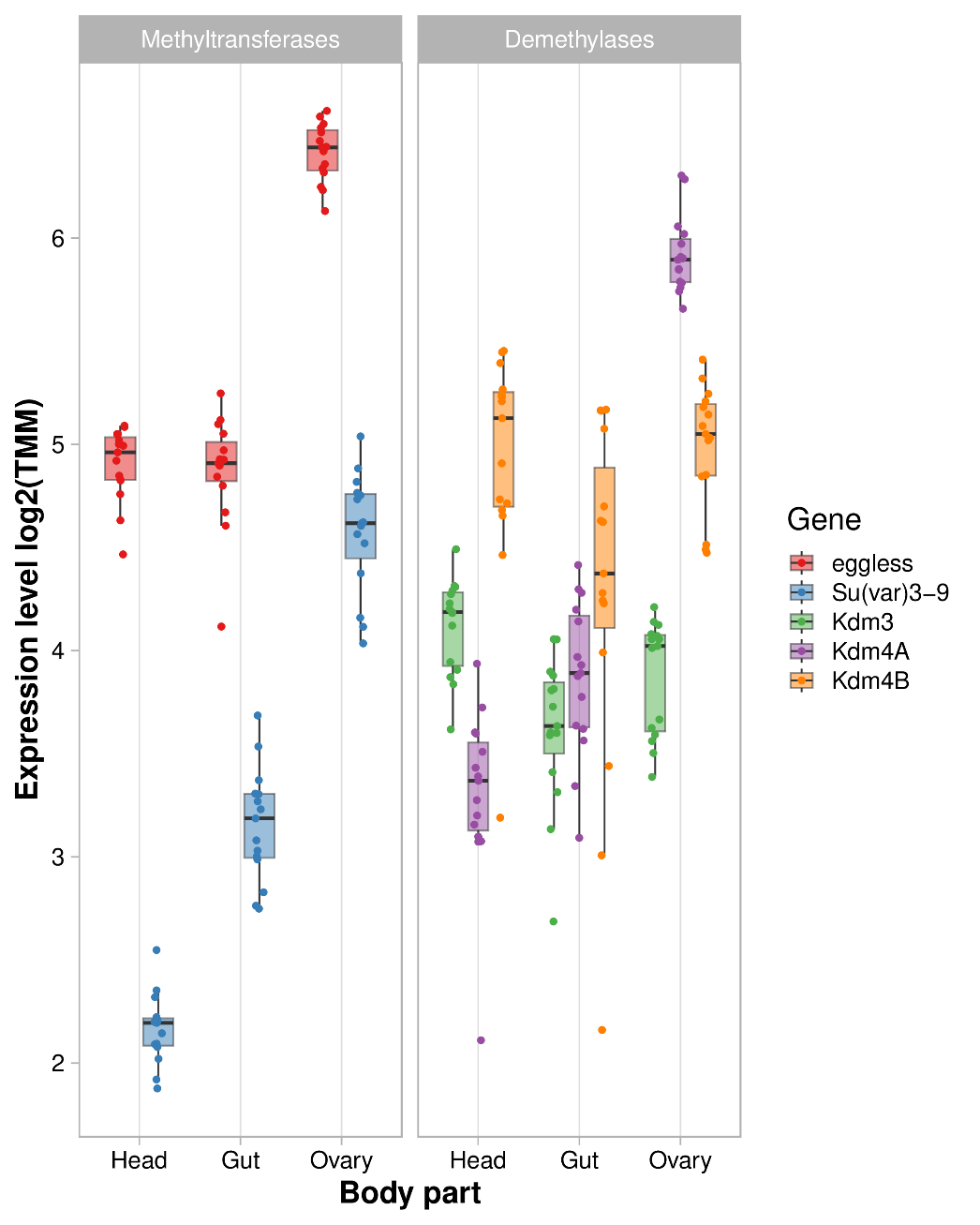


**Fig. S1. Expression level of methyltransferases and demethylases associated with H3K9me3 by body part.** Expression levels in log_2_ (TMM). Each observation corresponds to a replicate of each strain (in total 15 observations per gene).


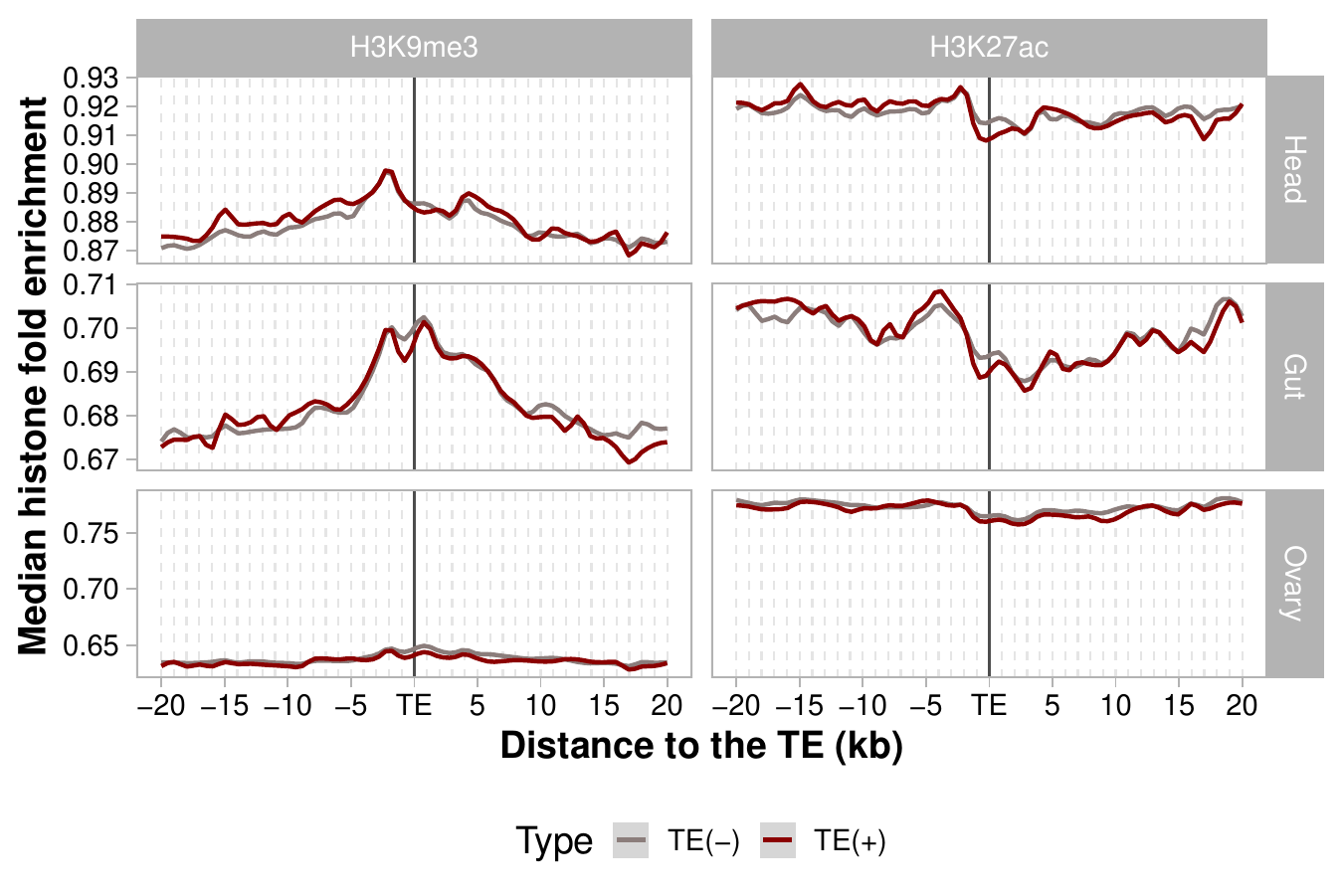


**Fig. S2. Median H3K9me3 and H2K27ac fold enrichment for “null” regions (*n*=5,175 regions).** Red line represents histone mark enrichment of genomes with a null region and the gray line represents histone mark enrichment of genomes without the null region. H3K9me3 and H3K27ac fold enrichment was averaged (median) over all sequences flanking the analyzed regions of all genomes. Plots were generated using LOESS smoothing (span = 10%).


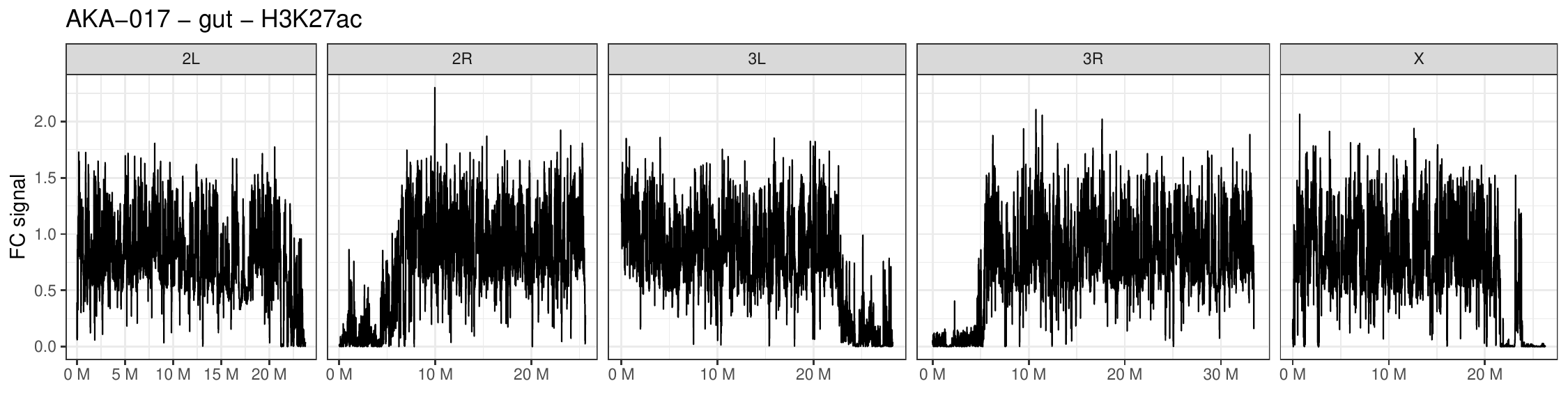

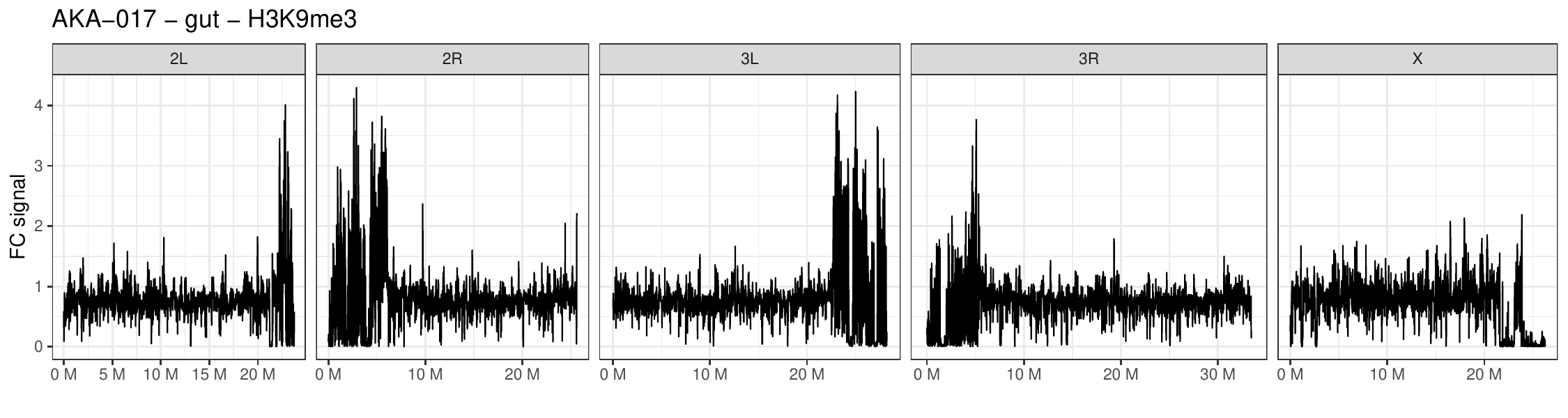

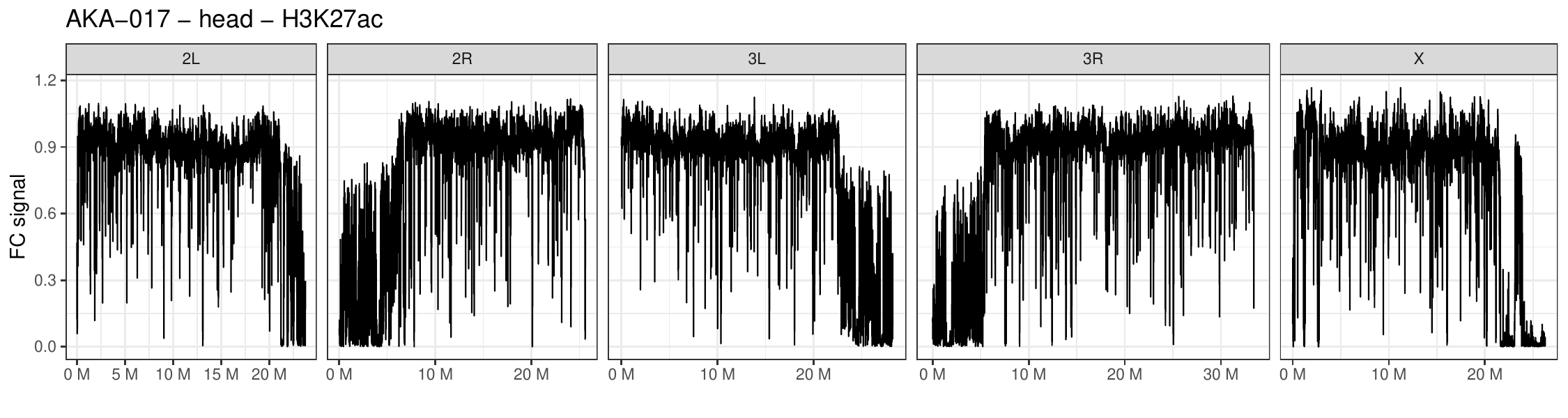

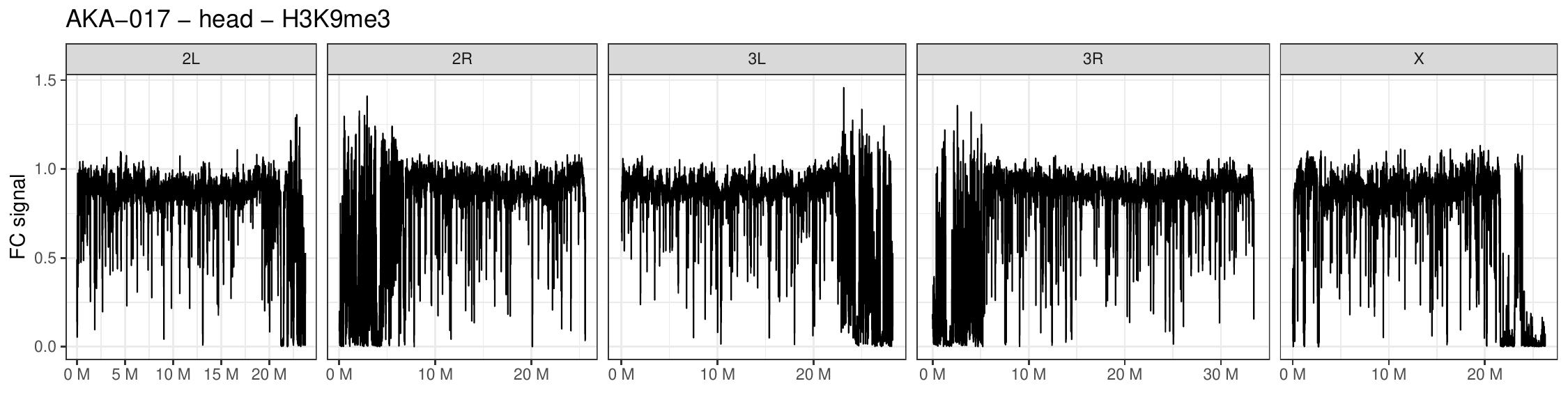

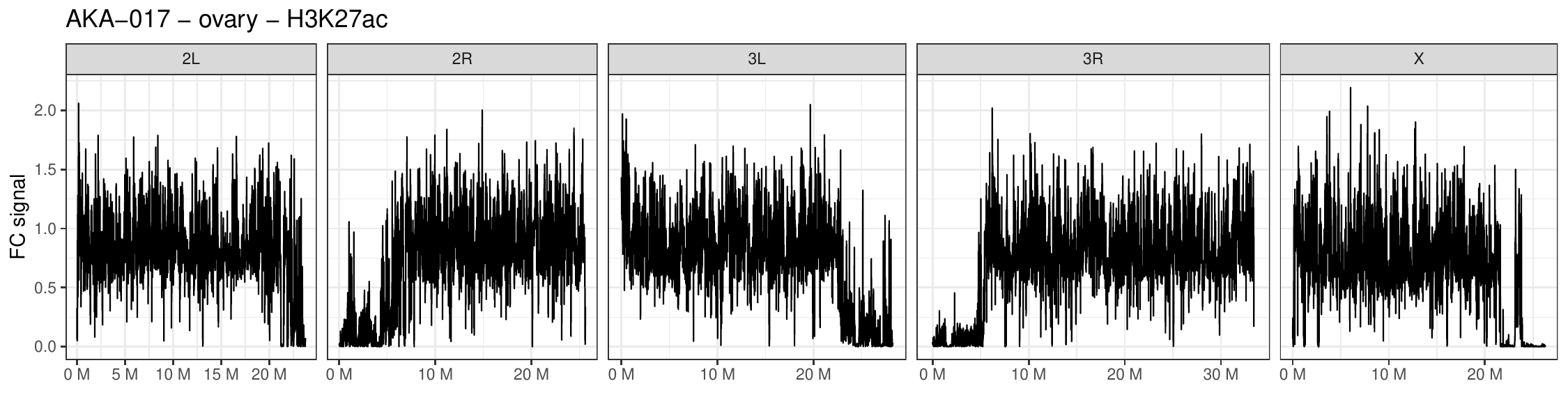

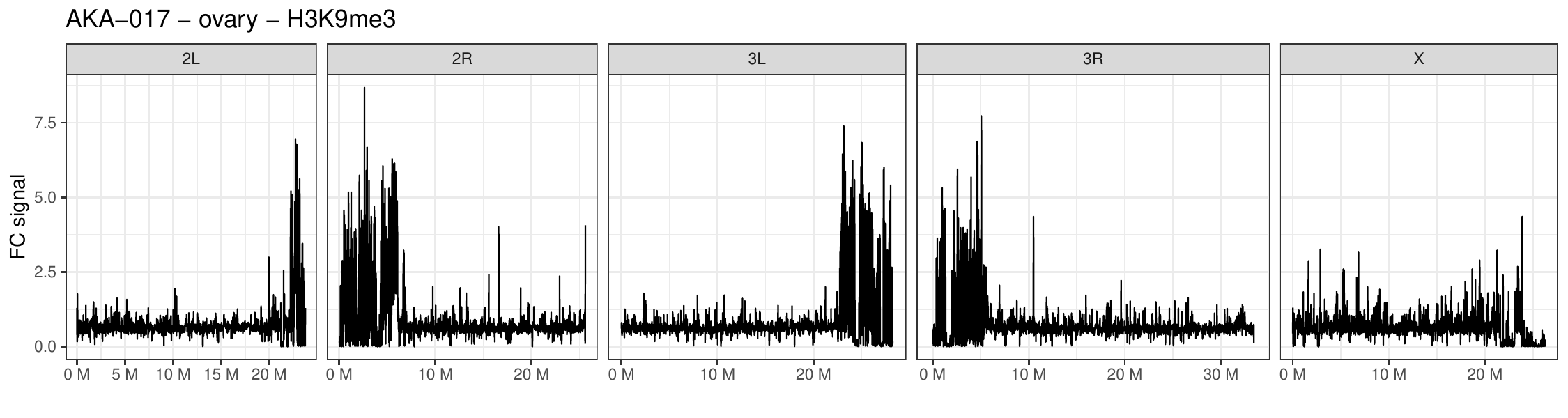


###### **
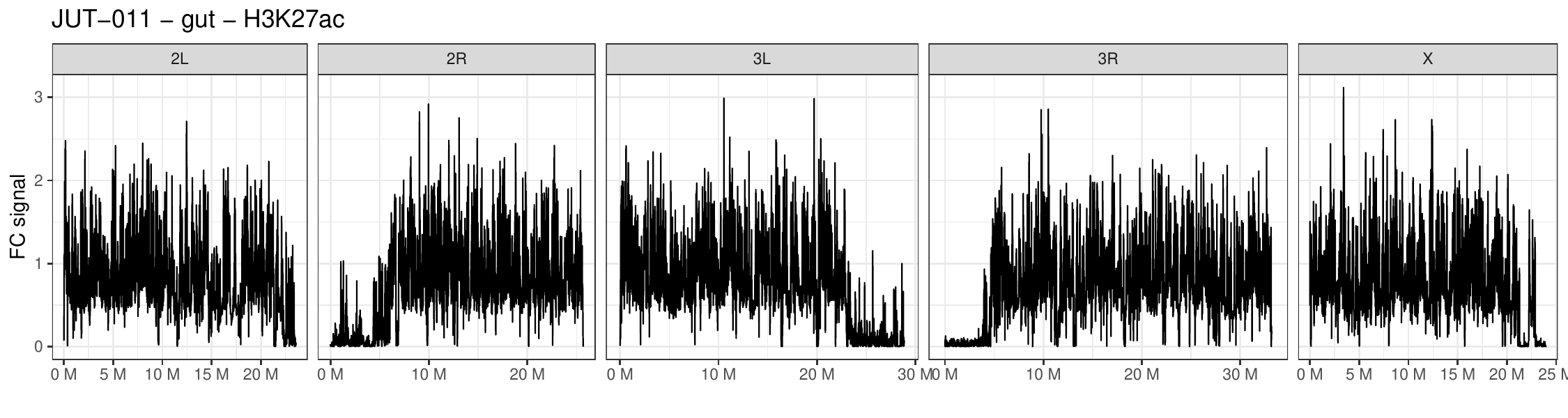

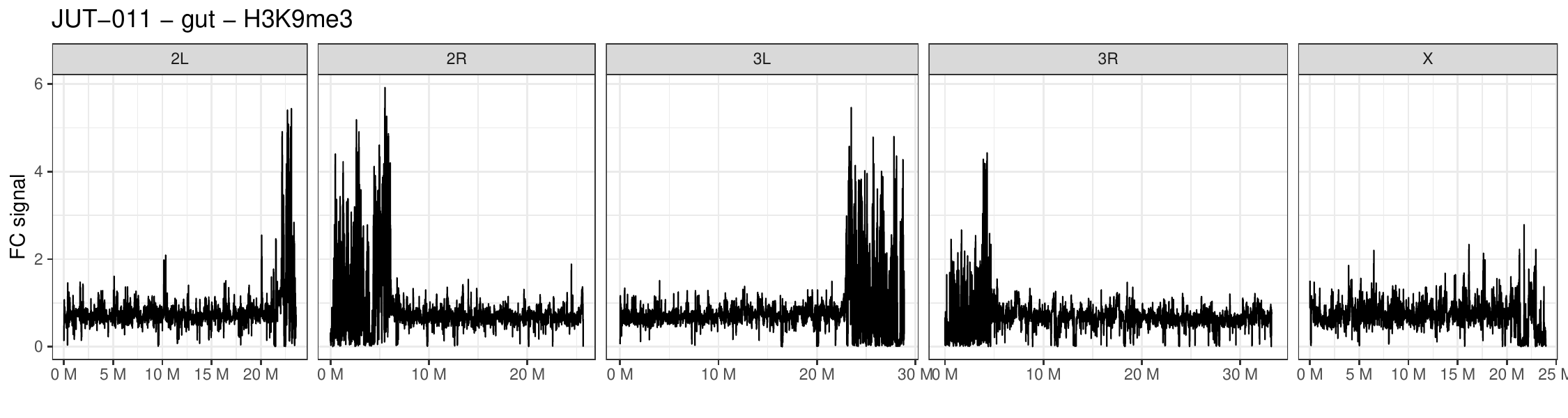

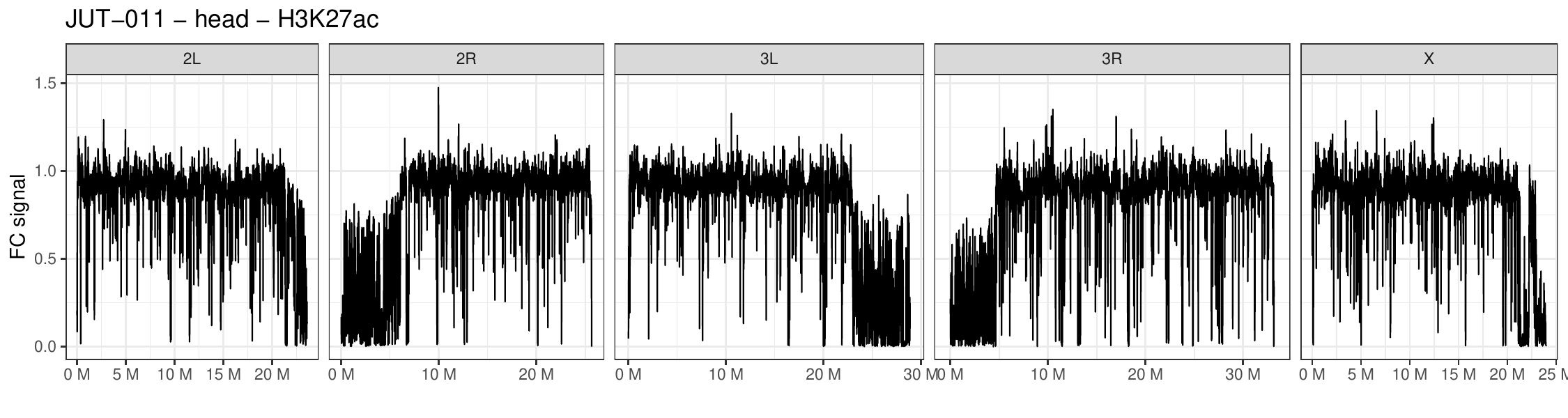

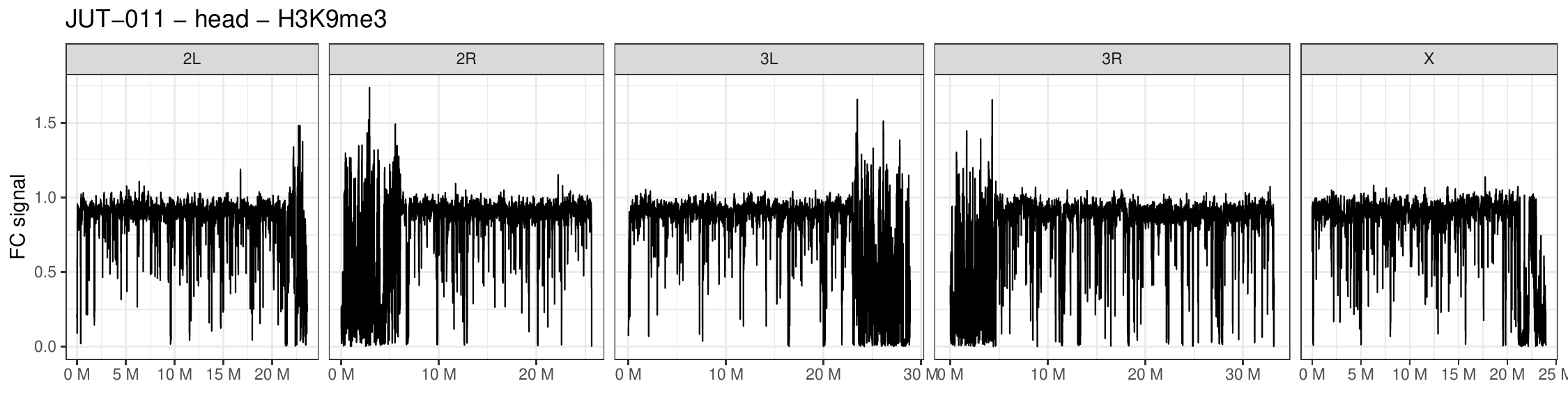

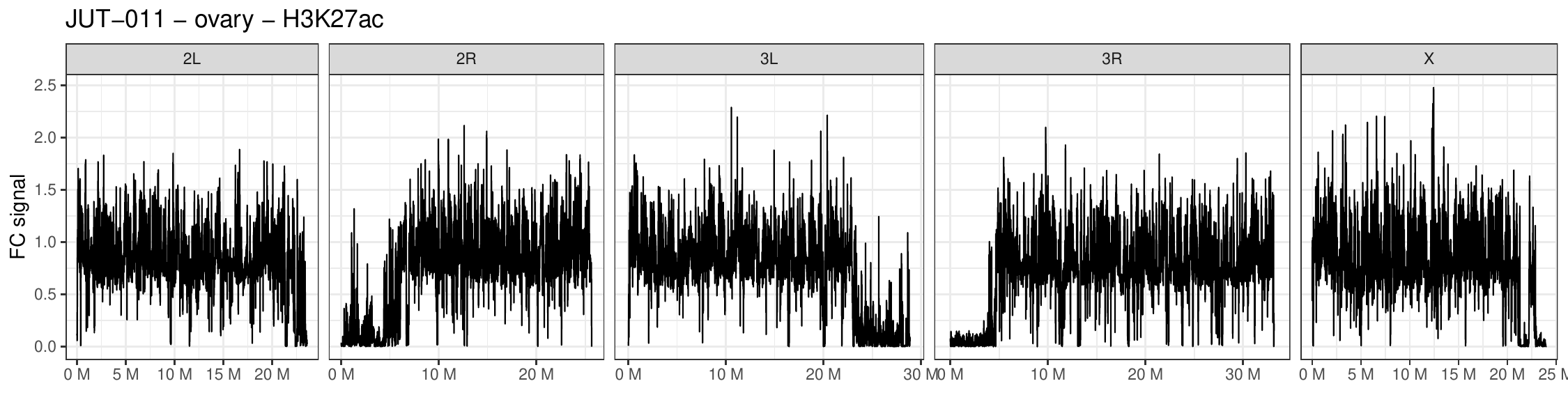

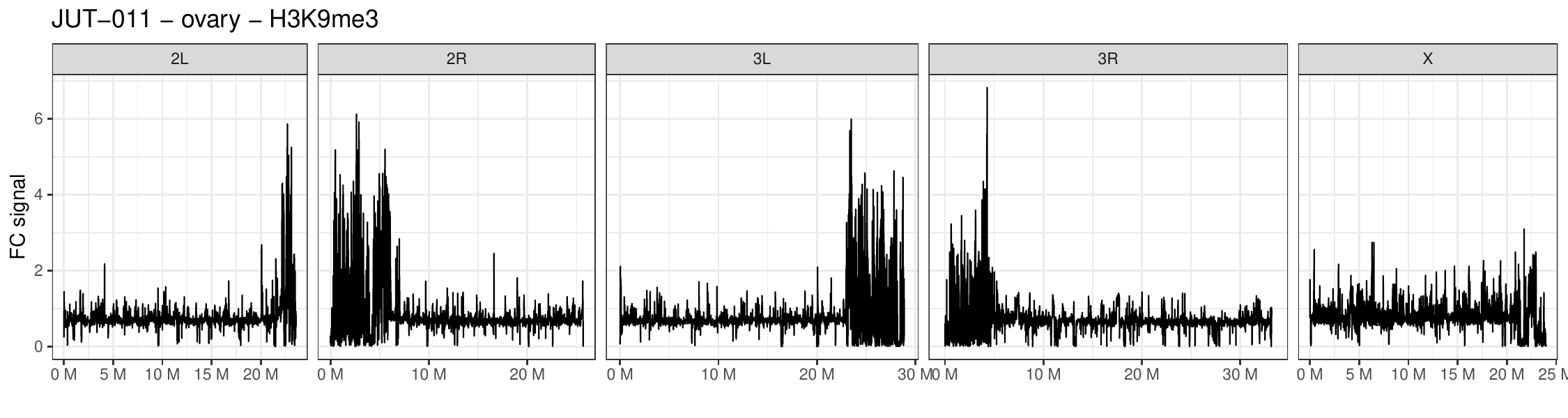
**

###### **
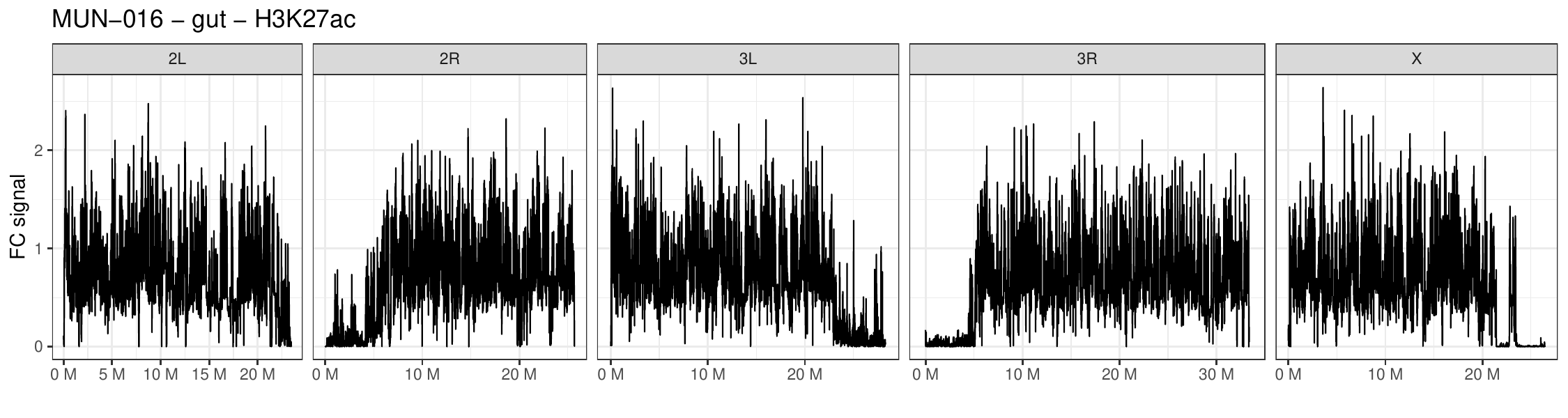

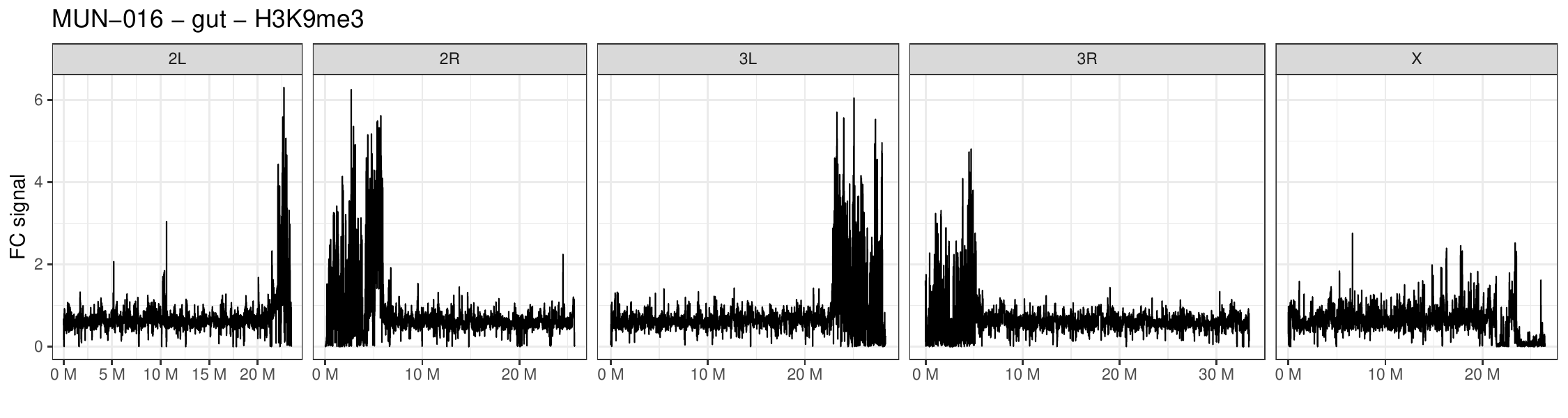

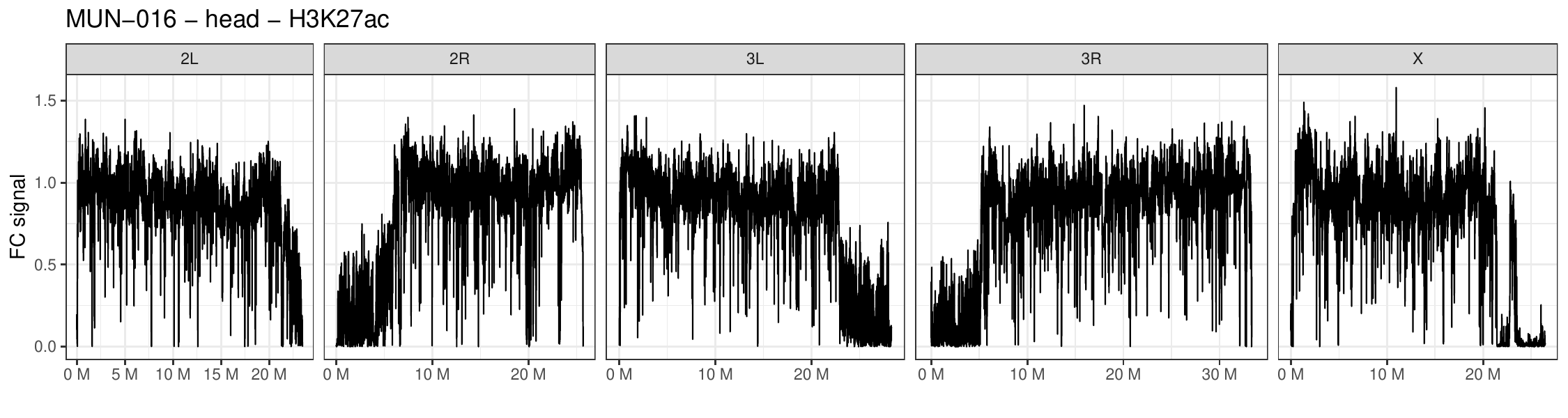

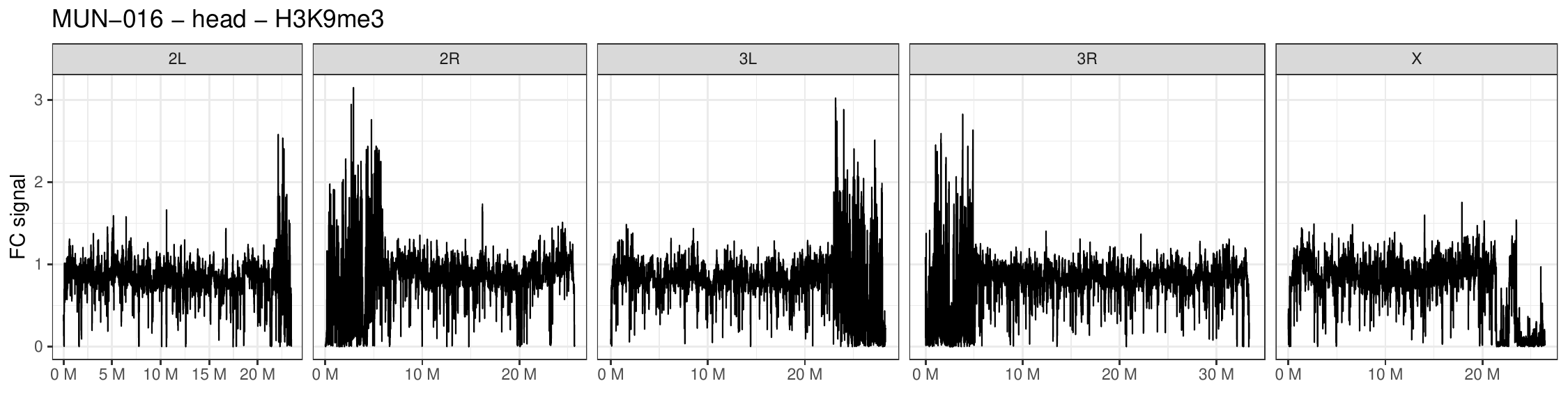

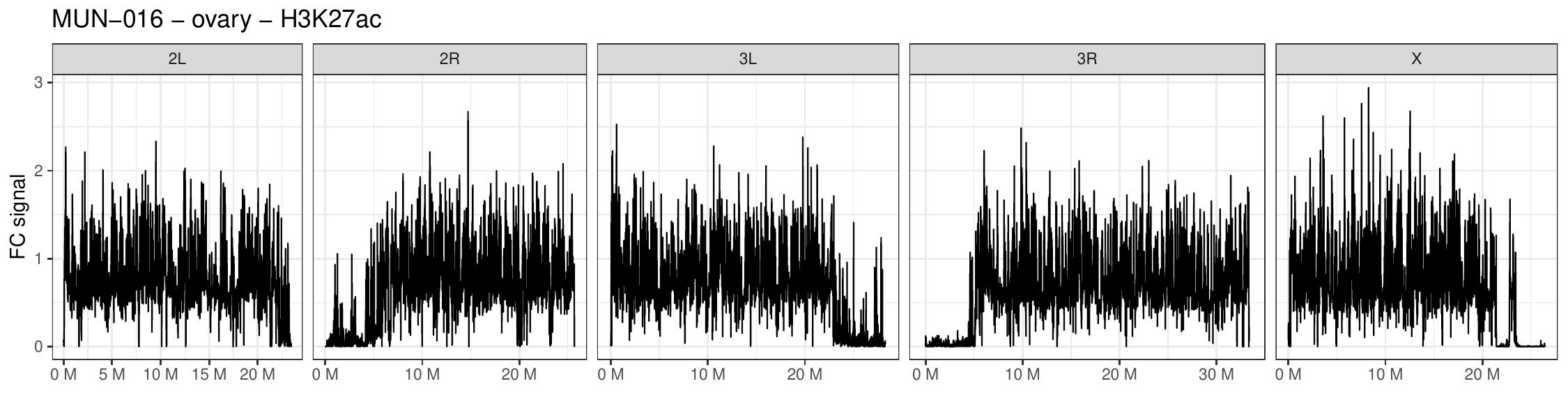

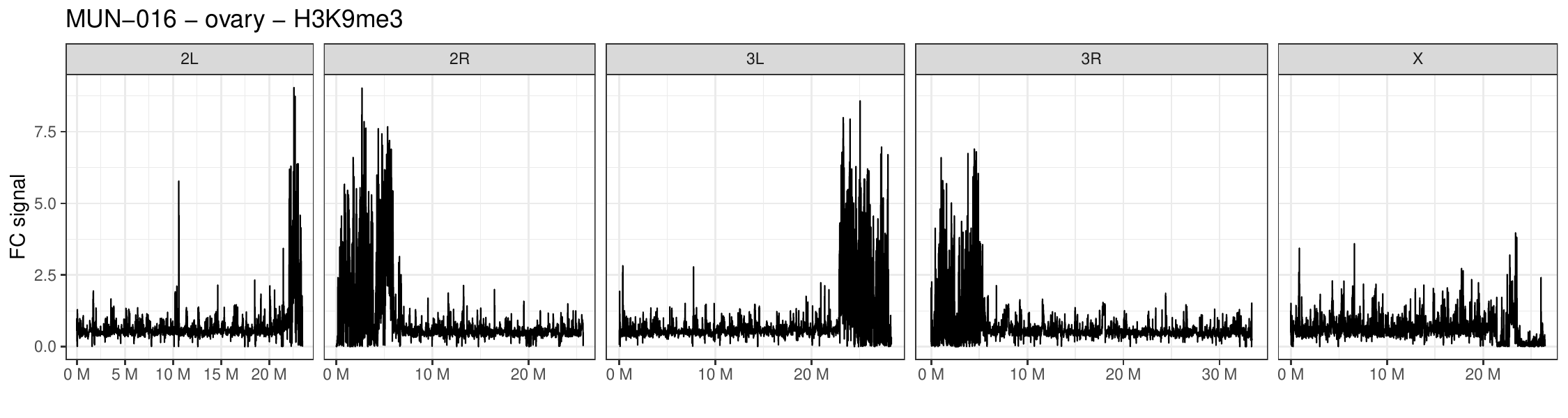
**

###### **
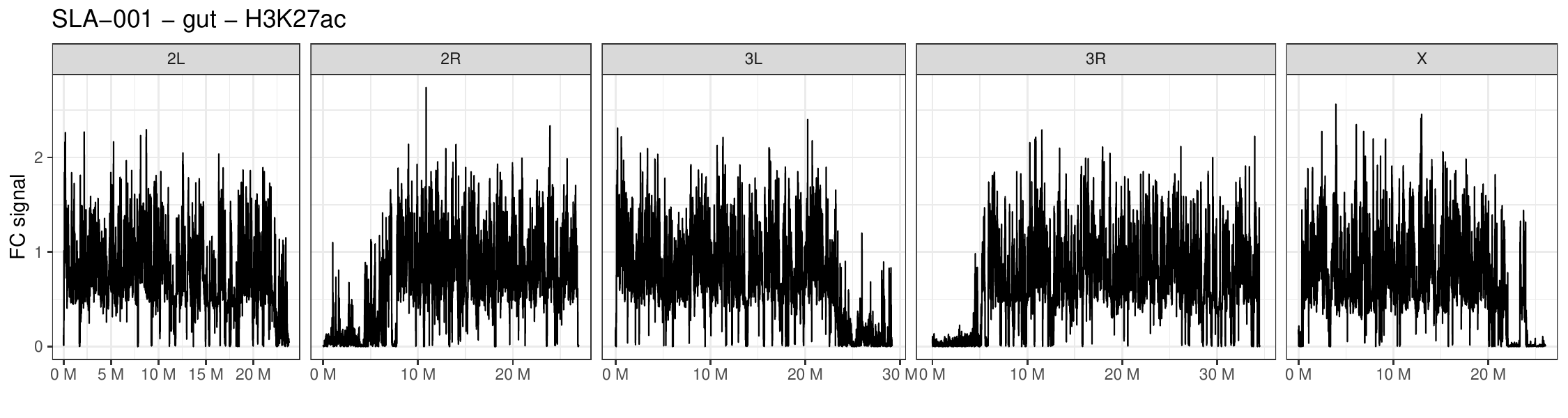

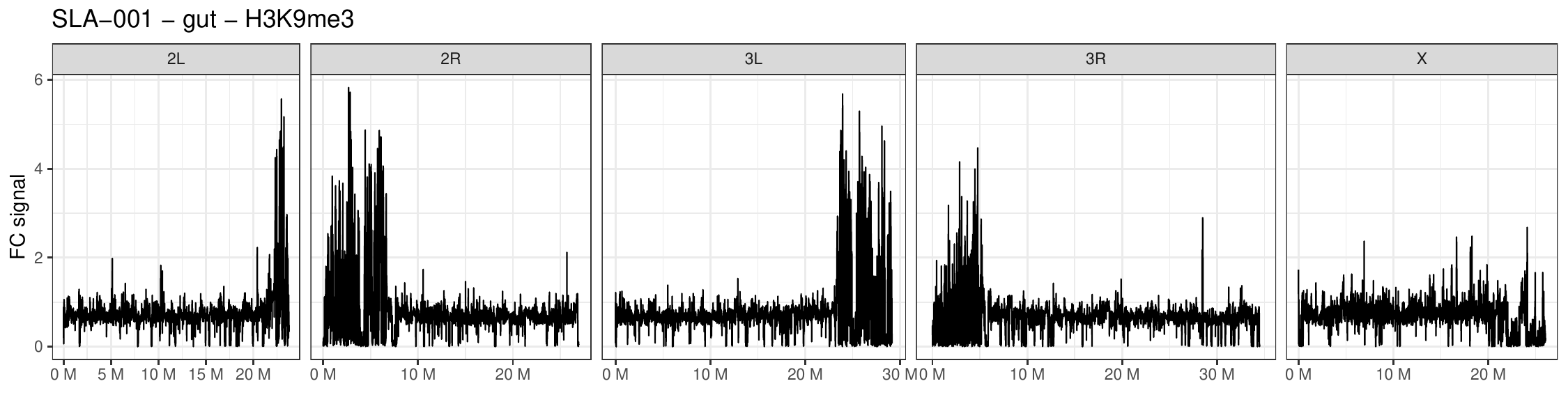

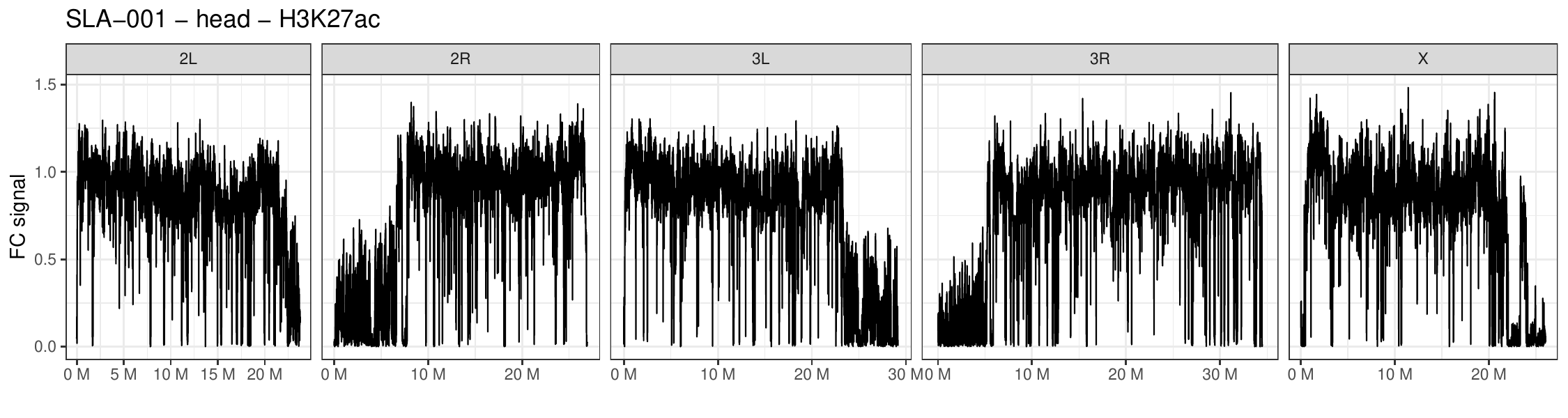

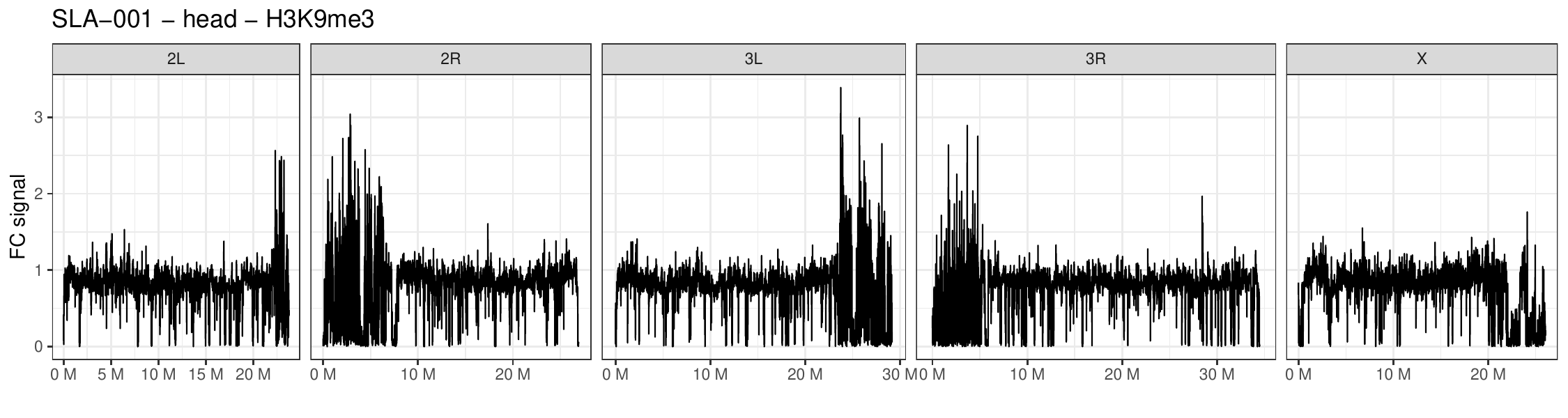

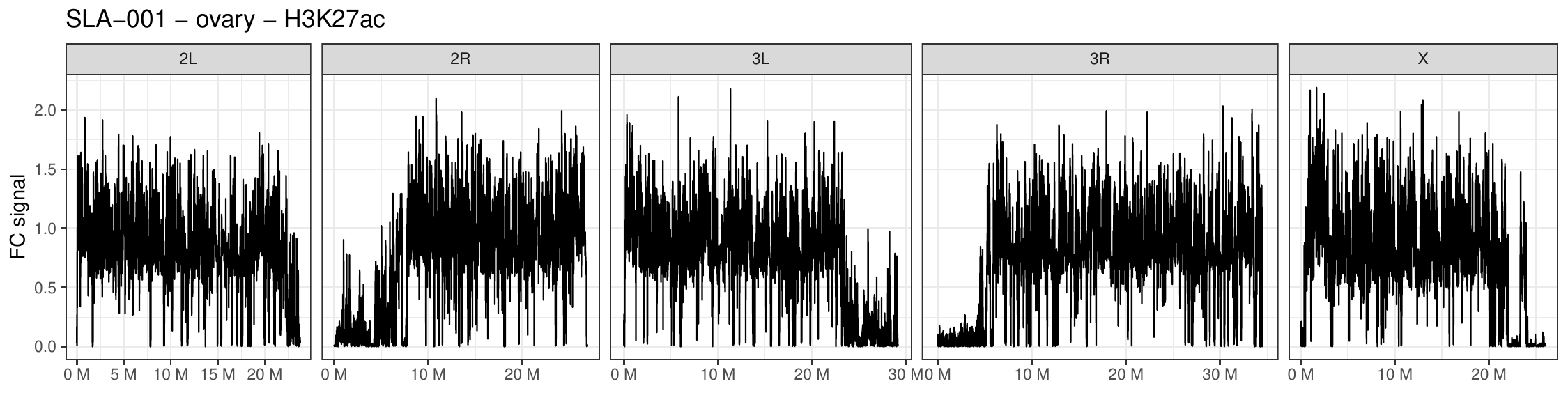

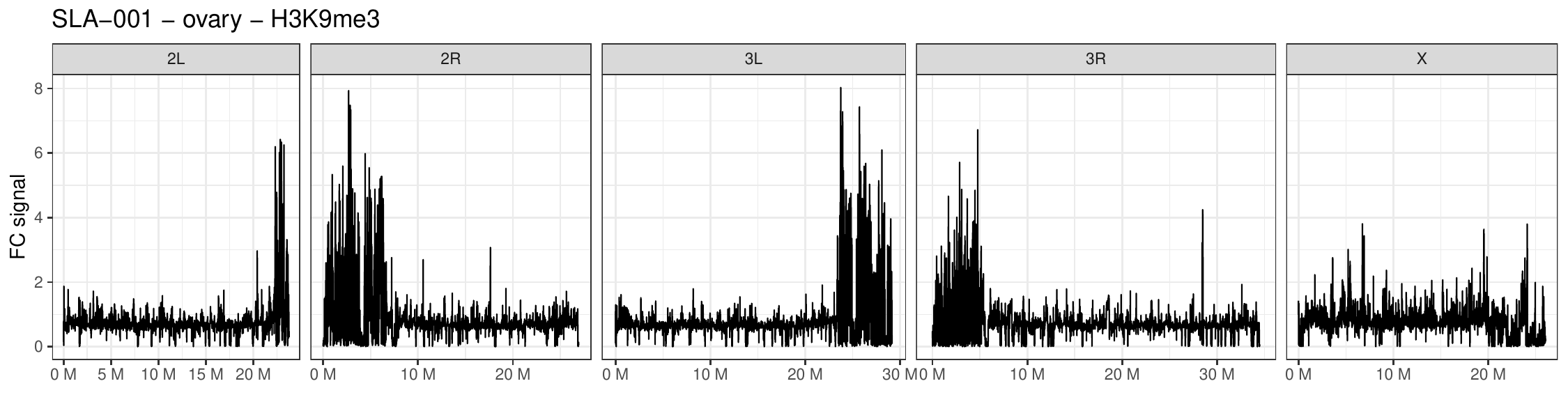
**

###### **
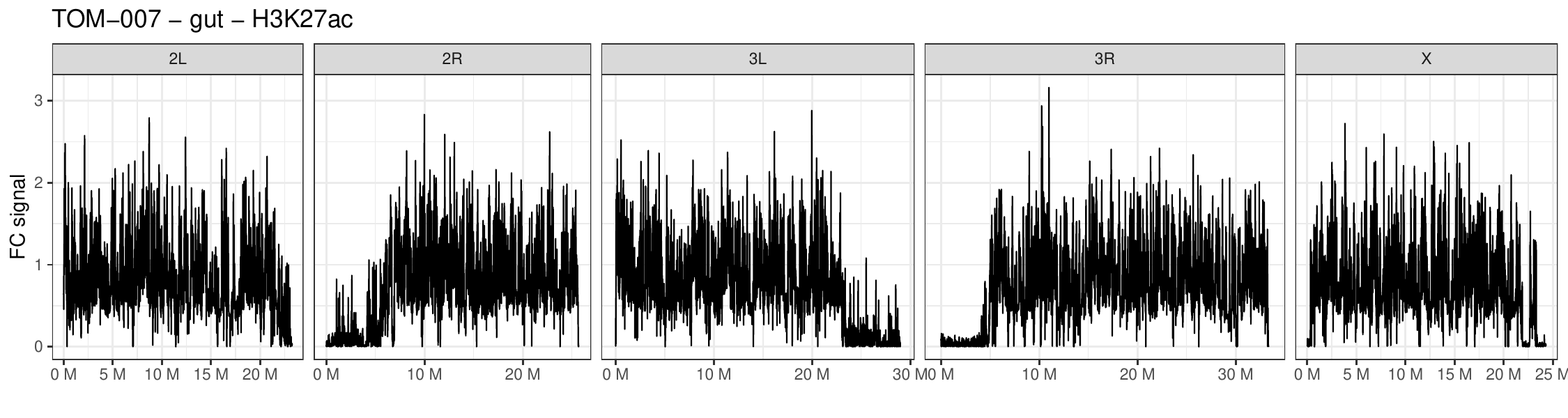

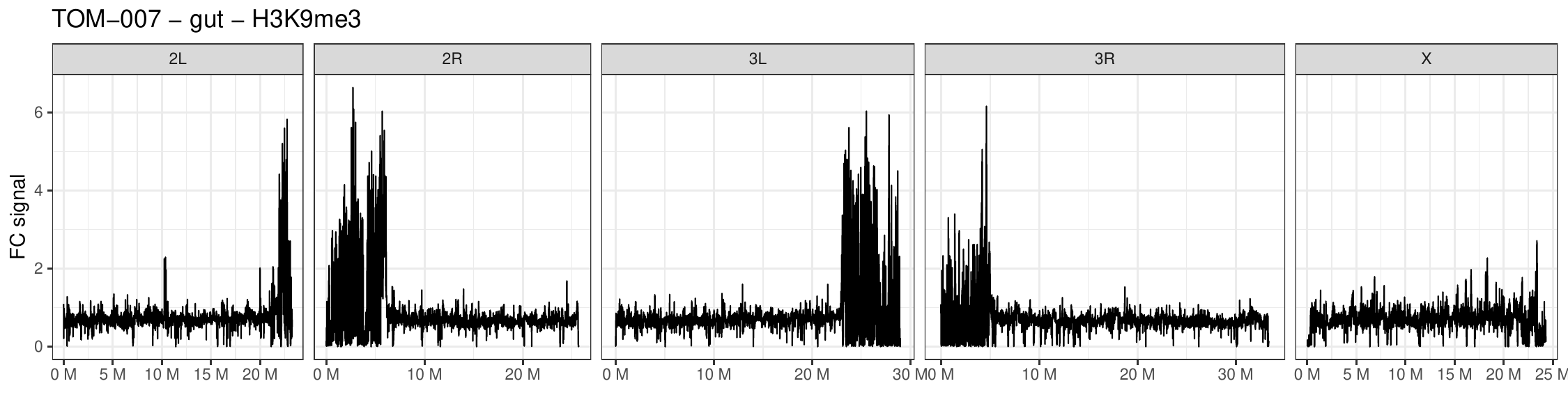

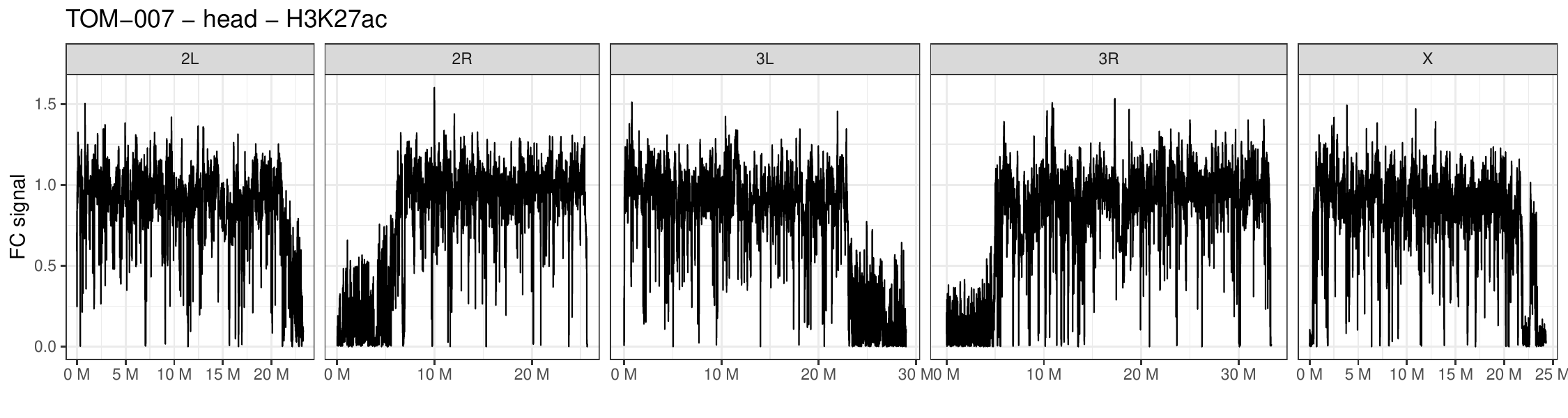

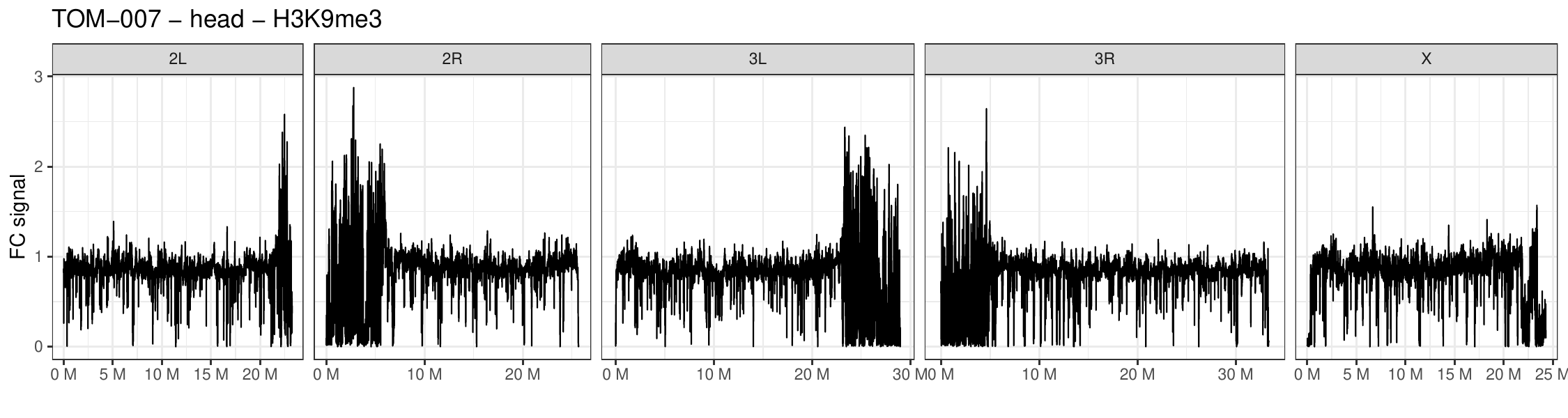

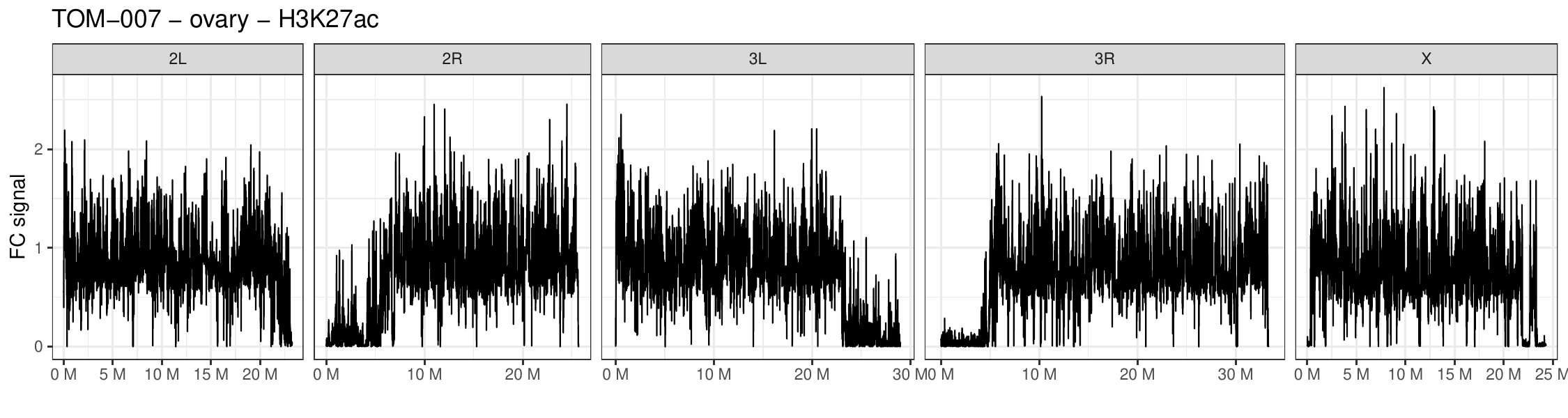

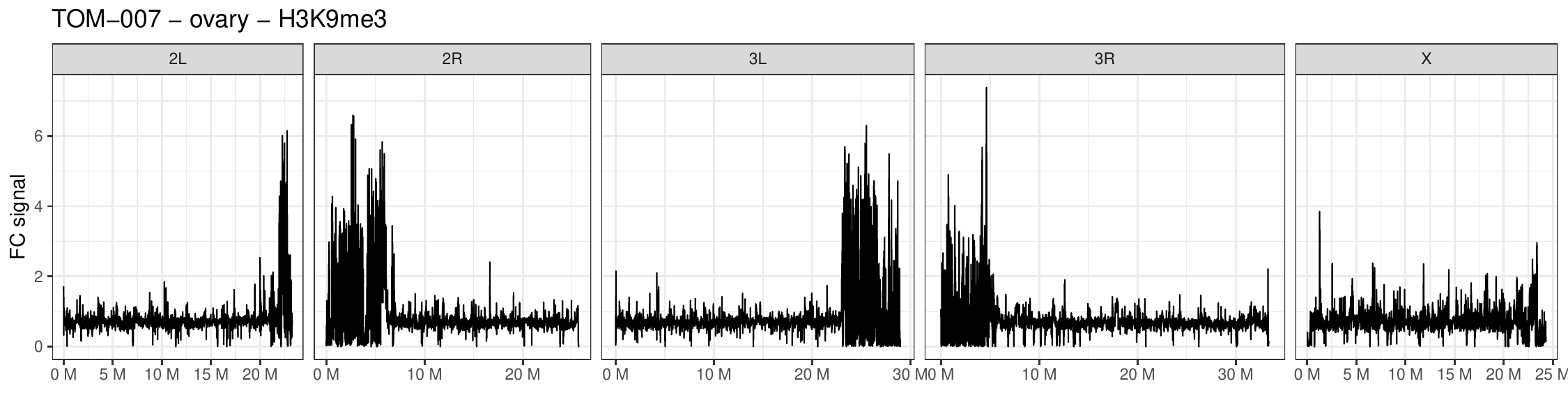
**

###### **Fig. S3.** Fold-enrichment signal calculated with MACS2, for each genome, body part and histone mark (30 sub-plots).

######


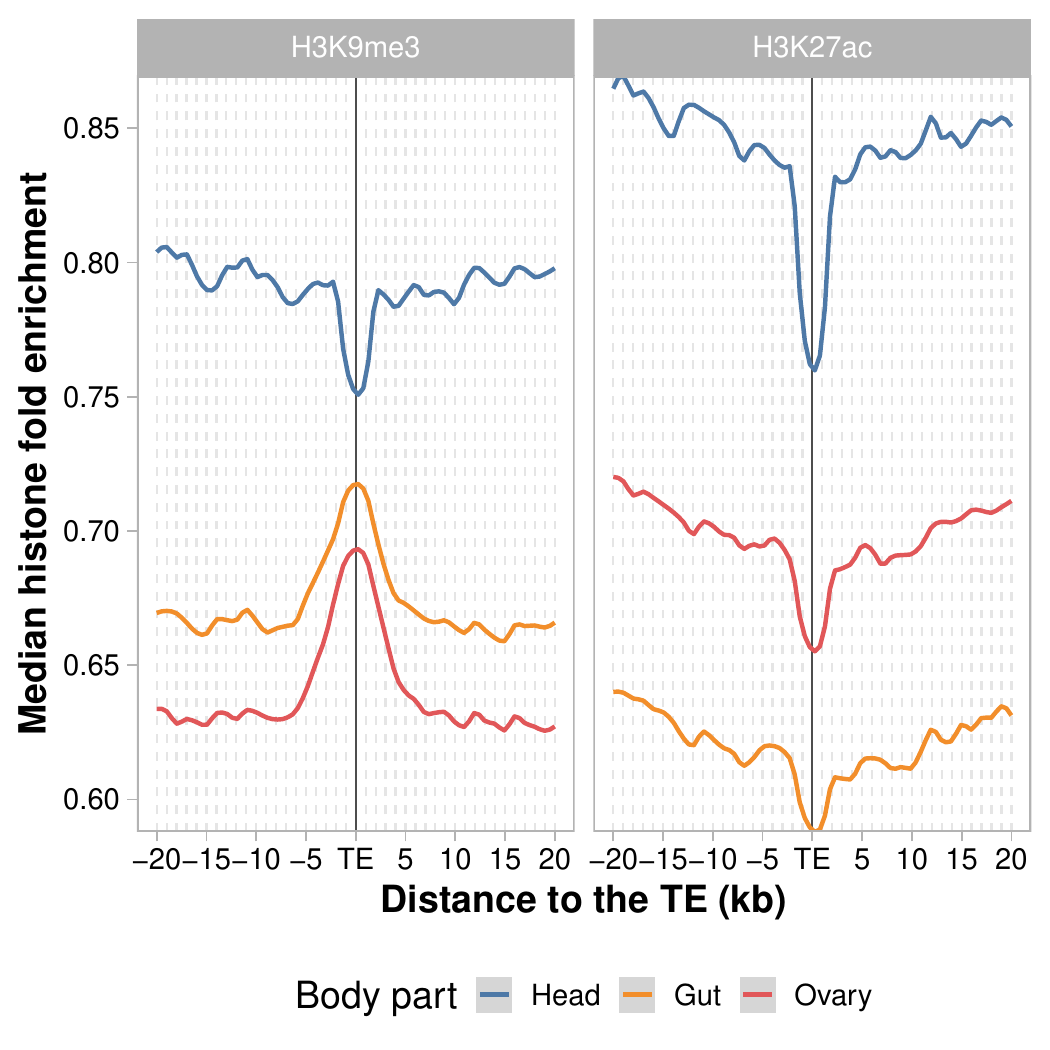


**Fig. S4. Epigenetic states in the ±20kb TEs flanking region across body parts. Removing AKA-017 and JUT-011 H3K9me3 and H3K27ac enrichment signal in head.** Median H3K9me3 and H3K27ac fold enrichment of all TEs annotated in five genomes (n=4,823 TEs), in head only considering 3 genomes (SLA-001, TOM-007 and MUN-016). Head (blue line), gut (orange line) ovary (red line).


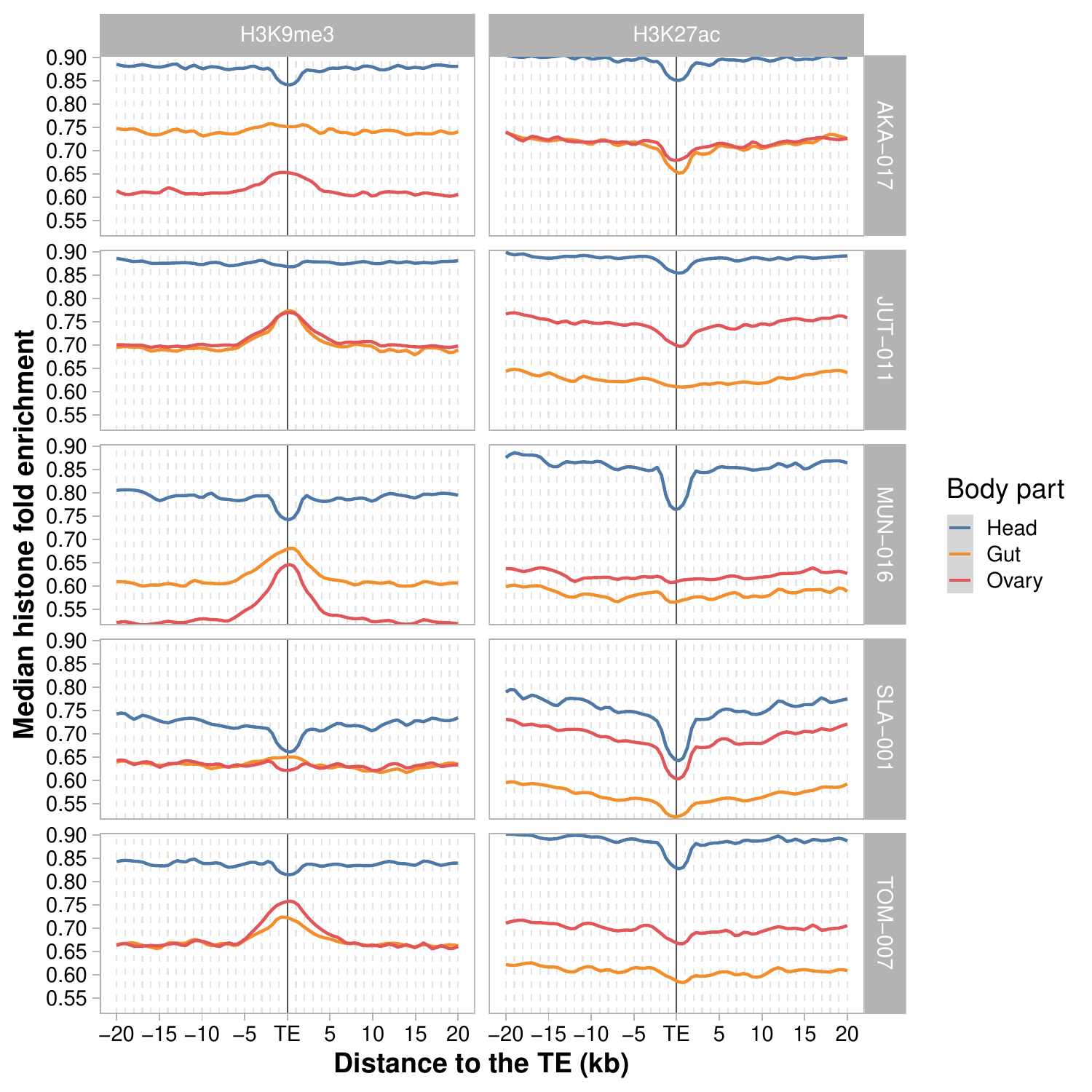


**Fig. S5 Epigenetic states in the ±20kb TEs flanking region across body parts by strain.** Median H3K9me3 and H3K27ac fold enrichment of all TEs annotated in five genomes (n=4,823 TEs). Head (blue line), gut (orange line) ovary (red line).

######
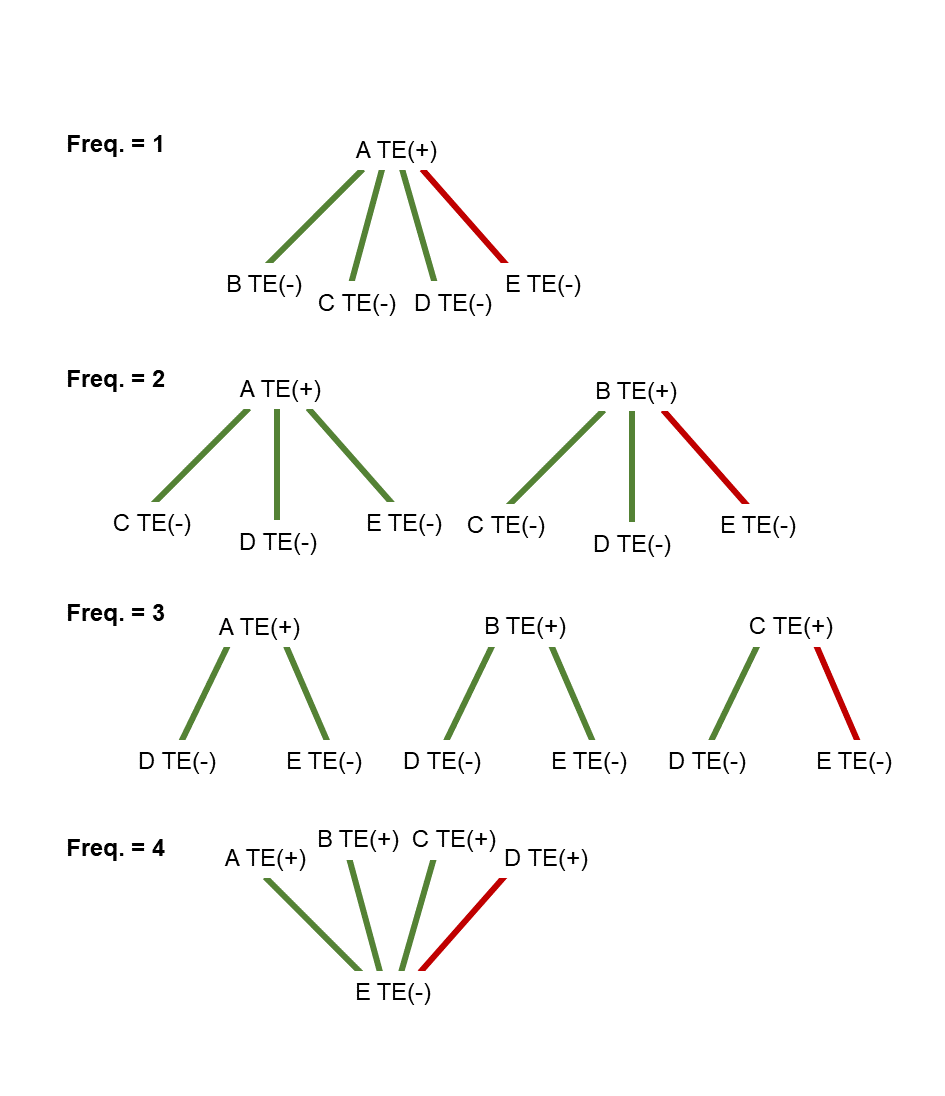


###### **Fig. S6.** To consider a TE with epigenetic effects we allowed only one different comparison across pairs of TE(+)-TE(-) genomes. A, B, C, D and E represent strains' names. TE(+) and TE(-) represent genomes with and without a TE insertion, respectively. Green lines indicate consistent comparisons, while red lines denote inconsistencies compared to the most prevalent change.

**
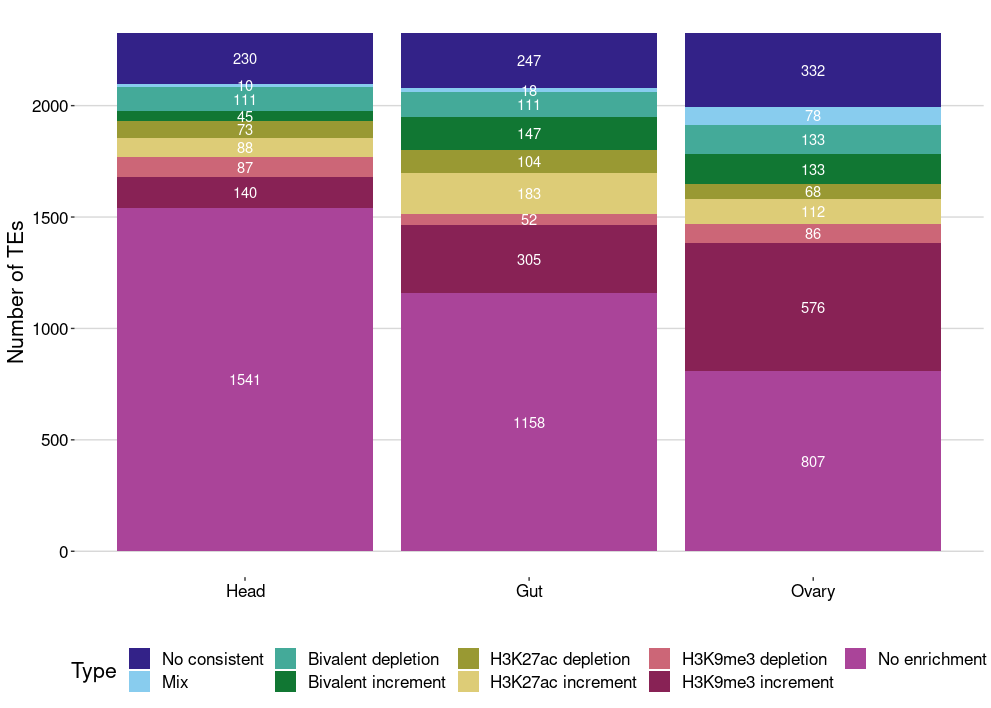
**

###### **Fig. S7.** Status of 2,325 polymorphic TEs across body parts.
